# The structural components of the *Azotobacter vinelandii* iron-only nitrogenase, AnfDKG, form a protein complex within the plant mitochondrial matrix

**DOI:** 10.1007/s11103-023-01363-3

**Published:** 2023-06-16

**Authors:** E. Johnston, S. Okada, C. M. Gregg, A. C. Warden, V. Rolland, V. Gillespie, K. Byrne, M. L. Colgrave, A. L. Eamens, R. S. Allen, C. C. Wood

**Affiliations:** 1 CSIRO Environment, GPO Box 1700, Acton, ACT 2601 Australia; 2 CSIRO Agriculture and Food, GPO Box 1700, Acton, ACT 2601 Australia; 3 CSIRO Agriculture and Food, 306 Carmody Rd, St Lucia, QLD 4067 Australia; 4 Australian Research Council Centre of Excellence for Innovations in Peptide and Protein Science, 306 Carmody Rd, St. Lucia, QLD 4067 Australia; 5 School of Environmental and Life Sciences, University of Newcastle, University Dr, Callaghan NSW 2308, Callaghan, Australia; 6 School of Health, University of the Sunshine Coast, Maroochydore, QLD 4558 Australia

**Keywords:** Nitrogenase, Protein interactions, Synthetic biology, Plant biotechnology, Mitochondrial engineering, Anf

## Abstract

A long-held goal of synthetic biology has been the transfer of a bacterial nitrogen-fixation pathway into plants to reduce the use of chemical fertiliser on crops such as rice, wheat and maize. There are three classes of bacterial nitrogenase, named after their metal requirements, containing either a MoFe-, VFe- or FeFe-cofactor, that converts N_2_ gas to ammonia. Relative to the Mo-nitrogenase the Fe-nitrogenase is not as efficient for catalysis but has less complex genetic and metallocluster requirements, features that may be preferable for engineering into crops. Here we report the successful targeting of bacterial Fe-nitrogenase proteins, AnfD, AnfK, AnfG and AnfH, to plant mitochondria. When expressed as a single protein AnfD was mostly insoluble in plant mitochondria, but coexpression of AnfD with AnfK improved its solubility. Using affinity-based purification of mitochondrially expressed AnfK or AnfG we were able to demonstrate a strong interaction of AnfD with AnfK and a weaker interaction of AnfG with AnfDK. This work establishes that the structural components of the Fe-nitrogenase can be engineered into plant mitochondria and form a complex, which will be a requirement for function. This report outlines the first use of Fe-nitrogenase proteins within a plant as a preliminary step towards engineering an alternative nitrogenase into crops.

**Supplementary Information:**

The online version contains supplementary material available at 10.1007/s11103-023-01363-3.

## Introduction

Optimal yields of many staple food crops, such as rice, wheat and maize, depend upon addition of nitrogen (N) fertiliser. Inert nitrogen gas (N_2_) is converted to ammonia (NH_3_) via the Haber–Bosch process, credited with supporting approximately half of the global population (Smil 1999). Essential to agriculture, the Haber–Bosch process accounts for approximately 2% of global energy use (Erisman et al. 2008). The use of N fertiliser also contributes to negative environmental consequences, including greenhouse gas production and fertiliser run-off, resulting in algal blooms and reduced water quality. Ideally, the global dependence on the fertiliser industry could be reduced via the development of new agricultural crops with lower N fertiliser requirements.

There are a number of bacteria and archaea, known collectively as diazotrophs, that contain nitrogenase, a unique enzyme complex that converts N_2_ to NH_3_ at ambient pressures and temperatures (Hogbom 2014; McRose et al. 2017). Nitrogenase is a two-component ATP-dependent metalloenzyme, with the catalytic component termed Component I, the dinitrogenase enzyme; and the ATP-dependent electron donor termed Component II, the dinitrogenase reductase enzyme. There are three known classes of nitrogenase that are named after the metal contained within the active-site cofactor of the catalytic component; the well-studied molybdenum-iron (Mo-) nitrogenase, and two so-called alternative nitrogenases where the molybdenum is replaced with vanadium or iron, named V- and Fe-nitrogenases, respectively (Davis et al. 1996; Robson et al. 1986). The genetics, biochemistry, and structural properties of the Mo-nitrogenase have been intensively studied (Buren and Rubio 2018; Dixon and Postgate 1972). The Mo-nitrogenase is encoded by *nif* genes. The structural elements of Component I, NifD and NifK, form a heterotetramer (α_2_β_2_). Component II is a homodimer of NifH. The alternative nitrogenases are less well characterised, although they are known to be both structurally and genetically related to Mo-nitrogenase. Unique components of the V- and Fe- systems are encoded by *vnf* and *anf* genes, respectively. Component I of these two nitrogenases consists of three proteins, VnfDKG or AnfDKG, respectively. The V- and recently resolved Fe-nitrogenase crystal structures have revealed heterohexamers, namely Vnf/AnfD_2_K_2_G_2_ (Sippel and Einsle 2017; Trncik et al. 2023). The VFe- or FeFe-cofactors essentially have the same structure as the FeMo-cofactor, apart from their respective signature metal that coordinates homocitrate. Both alternative nitrogenases encode a dinitrogenase reductase VnfH or AnfH, that form homodimers homologous to that of Component II (NifH) from the Mo-counterpart. Although Component II is commonly termed the obligate electron donor to Component I, it also serves other functions such as contributing to the assembly and maturation of the metalloclusters for Component I (Robinson et al. 1987; Allen et al. 1994; Burgess and Lowe 1996).

One ambitious option for reducing the current reliance on industrial N fertiliser is to genetically engineer the N fixation pathway into plants, enabling crops to produce their own biologically accessible N directly from the atmosphere (Buren et al. 2017; Good 2018). However, nitrogenases have demanding biochemical requirements including oxygen sensitivity, a need for reductant, ATP, and access to metals used in the assembly of the metalloclusters. These requirements need to be addressed to engineer the nitrogenase pathway in eukaryotes. We are exploring the mitochondrial matrix as a suitable location for the assembly and function of nitrogenases (Allen et al. 2017, 2020; Okada et al. 2020), an approach outlined in previous reviews (Curatti and Rubio 2014; Merrick and Dixon 1984). Encouragingly, some components required for assembly of the Mo-nitrogenase have been demonstrated to be functional when targeted to the yeast or plant mitochondrial matrix such as NifH (Lopez-Torrejon et al. 2016; Baysal et al. 2022; Jiang et al. 2021) and NifB (Buren et al. 2019, 2017). Additionally, the structural proteins of Mo-nitrogenase NifD and NifK have been demonstrated to form a heterotetramer within the yeast mitochondrial matrix, suggestive of the α_2_β_2_ structure found in their native bacterial hosts. Similarly, key proteins of the Fe-nitrogenase, AnfH, AnfD, AnfK, and AnfG, have been successfully expressed and isolated from yeast mitochondria (Lopez-Torrejon et al. 2021). This study established that (i) yeast AnfH was active *as isolated* for donation of electrons to NifDK and (ii) yeast AnfDK could be activated in vitro by addition of FeMo cofactor. Overall, these studies indicate that the mitochondrial matrix may be a location suitable for the expression, correct folding and assembly for components required to produce a functional Fe-nitrogenase.

Previously, we expressed 16 individual Nif proteins in plant mitochondria (Allen et al. 2017; Okada et al. 2020). Here we use a similar approach to simultaneously express four structural proteins of the Fe-nitrogenase, AnfDKGH, in the matrix of plant mitochondria. Encouragingly, we report that the introduced structural proteins AnfDKG interact within the mitochondrial matrix of *Nicotiana benthamiana* leaves.

## Materials and methods

### Generation of plant expression constructs

Plasmids for protein expression in plant cells were constructed using a modular cloning system with the Golden Gate assembly protocols based on Type IIS restriction enzymes (Peccoud et al. 2011). DNA components were commercially synthesised by Thermo Fisher Scientific or Twist Bioscience. Each construct used the same regulatory cassette, namely the 35S CaMV promoter (internal part number EC51288) and *Cauliflower mosaic virus* (CaMV) polyadenylation signal / terminator (EC41414). Each *anf* gene was commercially synthesised using a plant-derived codon bias for optimised expression, and where necessary duplicate sequences were designed to allow translational fusions at the N- and/or C-terminus. Plant codon optimized DNA parts *anfD* (EC38103, EC38106), *anfK* (EC38102), *anfH* (EC38100), *anfG* (EC38101), were used to construct SN and SL plasmids. The N-terminal translational fusion extensions allow targeting of proteins to the mitochondria or the cytosol. For mitochondrial targeting we used the first 51 amino acids of the Arabidopsis F1-ATPase subunit (pFAγ51) that included a well-characterised MPP cleavage site alone or followed by a HA epitope (EC38006 or EC38095 respectively), or the CoxIV mitochondrial targeting peptide including a processable MPP cleavage site followed by the Twin-Strep epitope (EC38091). Constructs were assembled into suitable backbone L1- acceptor vectors (EC47772, EC47742, EC47761, EC47781, EC47751) that includes standard TDNA based flanking regions using Type IIS restriction cloning with the BsaI enzyme for SN Golden Gate construction. SN constructs with the L1 acceptors were then assembled into multigene constructs using Type IIS restriction cloning, with the BbsI/BpiI enzyme for SL Golden Gate construction. The SN constructs were assembled onto a suitable SL acceptor plasmid (EC50505), with the Kanamycin resistance gene in position one on all SL constructs. All individual genes retained their own 35S CaMV promoter (part number EC51288) and CaMV polyadenylation signal / terminator (EC41414). The plasmid ID and descriptions are listed in Table 1 and 2 (single and multigene expression constructs, respectively). P19 and other standard constructs used as described previously (Wood et al. 2009).

**Table 1:** Summary of key features of Anf proteins expressed from single gene constructs used in this study

Predicted molecular weight (kDa)			
Plasmid	Expressed proteins	Full length	Processed*
SN81	pFAγ51-AnfD-HA	65.4	60.7
SN82	HA-AnfD	59.7	NA
SN129	pFAγ51-HA-AnfK	58.1	53.5
SN130	pFAγ51-HA-AnfH	36.9	32.4
SN131	pFAγ51-HA-AnfG	22.2	17.7
SN152	HA-AnfK	52.5	NA
SN153	HA-AnfH	31.3	NA
SN154	HA-AnfG	16.6	NA
SN155	alaFAγ51-HA-AnfK	57.4	NA
SN156	alaFAγ51-HA-AnfH	36.3	NA
SN157	alaFAγ51-HA-AnfG	21.6	NA
SN158	alaFAγ51-HA-AnfD	64.6	NA
SN161	pFAγ51-HA-AnfD	65.3	60.7
SN177	pFAγ51-AnfD-Twin-Strep	69.2	64.5
SN195	CoxIV-Twin-Strep-AnfK	57.7	54.7
SN227	pFAγ51-AnfD-HA	65.4	60.7
SN371	CoxIV-Twin-Strep-AnfG	21.9	18.9

All proteins expressed with 35S promoter and terminator. Nucleotide and corresponding amino acid sequences of the genes are listed in Supplementary Data Set 3

^*^Processed refers to the molecular weight after cleavage of the MTP by the MPP within the mitochondrial matrix. NA, not applicable. HA and Twin-Strep are tags, pFAγ51 and CoxIV are mitochondrial targeting peptides

**Table 2:** Summary of key features of Anf proteins expressed from multigene constructs used in this study

Predicted molecular weight (kDa)			
Plasmid	expressed proteins	Full length	Processed*
SL26	pFAγ51-HA-AnfG	22.2	17.7
	pFAγ51-HA-AnfD	65.3	60.7
	pFAγ51-HA-AnfK	58.1	53.5
	pFAγ51-HA-AnfH	36.9	32.4
SL27	pFAγ51-HA-AnfD	65.3	60.7
	pFAγ51-HA-AnfK	58.1	53.5
	pFAγ51-HA-AnfH	36.9	32.4
SL28	pFAγ51-HA-AnfD	65.3	60.7
	pFAγ51-HA-AnfK	58.1	53.5
SL29	pFAγ51-HA-AnfD	65.3	60.7
SL31	alaFAγ51-HA-AnfD	64.6	NA
	alaFAγ51-HA-AnfK	57.4	NA
	alaFAγ51-HA-AnfH	36.3	NA
	alaFAγ51-HA-AnfG	21.6	NA
SL37	pFAγ51-HA-AnfG	22.2	17.7
	pFAγ51-AnfD-HA	65.4	60.7
	CoxIV-Twin-Strep-AnfK	57.7	54.7
	pFAγ51-HA-AnfH	36.9	32.4
SL92	pFAγ51-HA-AnfG	22.2	17.7
	pFAγ51-HA-AnfD	65.3	60.7
	pFAγ51-HA-AnfK	58.1	53.5
SL93	CoxIV-Twin-Strep-AnfG	21.9	18.9
	pFAγ51-HA-AnfK	58.1	53.5
	pFAγ51-HA-AnfD	65.3	60.7
	pFAγ51-HA-AnfH	36.9	32.4

All proteins expressed with 35S promoter and terminator

^*^Processed refers to the molecular weight after cleavage of the MTP by the MPP within the mitochondrial matrix. NA, not applicable. HA and Twin-Strep are tags, pFAγ51 and CoxIV are mitochondrial targeting peptides

### Growth of *Agrobacterium* and protein expression in leaves of *N. benthamiana*

*Nicotiana benthamiana* plants were grown in a Conviron™ growth chamber at 23 °C under a 16:8 h light: dark cycle with 90 µmol/min light intensity provided by cool white fluorescent lamps. *Agrobacterium tumefaciens* strain GV3101 (SN or SL constructs) or AGL1 (P19 construct) cells were grown to stationary phase at 28 °C in LB broth supplemented with 50 mg/L carbenicillin or 50 mg/L kanamycin, according to the selectable marker gene on the construct, 25 mg/L gentamycin and 50 mg/L rifampicin. Acetosyringone was added to the culture to a final concentration of 100 μM and the culture then incubated at 28 °C with shaking for another 2.5 h. The bacteria were then pelleted by centrifugation at 5000 x*g* for 10 min at room temperature. The supernatant was discarded, and the pellet was resuspended in 10 mM MES pH 5.7, 10 mM MgCl_2_ and 100 μM acetosyringone (infiltration buffer) after which the OD_600_ was measured. Each culture was added to a fresh tube at OD_600_ = 0.10, and the final volume was made up with the infiltration buffer. In gene combinations where multiple T-DNA/Agrobacteria were mixed, each agrobacterium was added to the infiltration mix at a final OD_600_ of 0.1. Leaves of five-week-old plants were then infiltrated with the culture mixture and the plants were grown for four or five days before leaf discs were harvested during the light period for analysis.

### Protein extraction and analysis by western blot

Infiltrated *N. benthamiana* leaves were harvested as leaf discs with a diameter of 1.5 cm, proteins extracted, and protein expression detected by western blot (Allen et al. 2017). The HA antibody (Monoclonal Anti-hemagglutinin (HA), Sigma) was used at 1:5000 dilution, and the anti-Strep/horseradish peroxidase (HRP) conjugate antibody (Strep-MAB-conjugate HRP, IBA Lifesciences) was used at 1:10,000. For testing the solubility of plant-expressed Anf proteins the leaf tissue was ground in ice-cold extraction buffer (100 mM Tris pH 8.0, 150 mM NaCl, 0.25 M mannitol, 5% (v/v) glycerol, 1% (v/v) Tween 20, 1% (w/v) polyvinylpyrrolidone 40, 2 mM Tris(2-carboxyethyl)phosphine hydrochloride, 0.2 mM phenylmethylsulfonyl fluoride, 10 µM leupeptin) and transferred to a microfuge tube. The sample was centrifuged at 20,000 x*g* for 5 min and separated into soluble (supernatant) and insoluble (pellet) fractions. The supernatant was transferred to a fresh microfuge tube and centrifuged again at 20,000 x*g* for 5 min. The insoluble fraction was washed three times by resuspension of the pellet in 1 mL extraction buffer. For each cycle the pellet was dispersed in extraction buffer by repeated pipetting followed by centrifugation at 20,000 x*g* for 5 min. This cycle was repeated twice more. Laemmli buffer was added to the resulting soluble and insoluble fractions and subjected to sodium dodecyl sulfate–polyacrylamide gel electrophoresis (SDS-PAGE) followed by western blot analysis (Allen et al. 2017; Okada et al. 2020). Samples and molecular weight markers (BenchMark™ or PageRuler™ pre-stained protein ladder, Thermo Fisher Scientific) were electrophoresed on 4–12% NuPage™ Bis–Tris protein gels (Thermo Fisher Scientific) for 60 min at 200 V, using 20 μL of sample per lane. Proteins were blotted to PVDF membrane using an iBLOT apparatus (Thermo Fisher Scientific) and proteins containing an epitope detected by using anti-HA or anti-Strep antibodies.

### Proteomic analysis by liquid chromatography-tandem mass spectrometry (LC–MS/MS)

#### Tryptic digestion

A variety of protein sources expressed from single- and multigene constructs (Table 1 and 2) were used for liquid chromatography-tandem mass spectrometry (LC–MS/MS) analysis. Protein samples were prepared either in-solution or as excised gel slices. The in-gel digests were prepared as described previously (Colgrave et al. 2019). The solutions were subjected to the filter-aided sample preparation (FASP), a method used for the on-filter digestion of proteins prior to mass spectrometry-based analyses (Wisniewski et al. 2009). In brief, 100 μL (~ 200 μg) of protein was diluted in 8 M urea, 100 mM Tris–HCl, pH 8.5 (UA buffer) and applied to a 10 kDa molecular weight cut-off filter (Merck Millipore, Australia). The filters were centrifuged (20,800 × *g* for 15 min) and washed with 200 μL of UA buffer. The total protein extracts were reduced with 50 mM dithiothreitol (DTT) and the mixture incubated at room temperature (~ 20–22 °C) for 50 min with shaking. The filters were centrifuged (20,800 × *g*, 15 min) and washed with two 200 μL volumes of 8 M urea, 100 mM Tris–HCl, pH 8.5. For cysteine alkylation, 100 μL of 50 mM iodoacetamide in 8 M urea, 100 mM Tris–HCl was added and the mixture incubated at RT in the dark for 30 min. The filters were centrifuged (20,800 × *g*, 15 min) to remove excess iodoacetamide and washed with two 200 μL volumes of 8 M urea, 100 mM Tris–HCl. The buffer was exchanged using 50 mM ammonium bicarbonate (pH 8.5) by two consecutive wash/centrifugation steps. Trypsin (Promega, Alexandria, Australia) solution (200 μL, 20 μg/mL in 50 mM ammonium bicarbonate, 1 mM CaCl_2_) was added and the mixture incubated overnight at 37 °C. The 10 kDa filters were transferred to fresh collection tubes and washed with two volumes of 200 μL of 50 mM ammonium bicarbonate by centrifugation (20,800 xg, 15 min) and the filtrates combined and lyophilised. The tryptic peptides were resuspended in 50 μL of 1% formic acid and stored at 4 °C until LC–MS/MS analysis.

#### Global proteomic profiling

The trypsin digested peptides from in-solution digests were analyzed using chromatographic separation (4 μL) on an Ekspert nanoLC415 (Eksigent, Dublin, CA, U.S.A.) directly coupled to a TripleTOF 6600 l chromatography tandem mass spectrometer (SCIEX, Redwood City, CA, USA). The peptides were desalted for 5 min on a ChromXP C18 (3 μm, 120 Å, 10 mm × 0.3 mm) trap column at a flow rate of 10 μL/min 0.1% FA, and separated on a ChromXP C18 (3 μm, 120 Å, 150 mm × 0.3 mm) column at a flow rate of 5 μL/min at 30 °C. A linear gradient from 3–25% solvent B over 68 min was employed followed by: 5 min from 25% B to 35% B; 2 min 35% B to 80% B; 3 min at 80% B, 80–3% B, 1 min; and 8 min re-equilibration. The solvents were: (A) 5% dimethylsulfoxide (DMSO), 0.1% formic acid (FA), 94.9% water; (B) 5% DMSO, 0.1% FA, 90% acetonitrile, 4.9% water. The instrument parameters were: ion spray voltage 5500 V, curtain gas 25 psi, GS1 15 psi and GS2 15 psi, heated interface 150 °C. Data were acquired in information-dependent acquisition mode comprising a time-of-flight (TOF)-MS survey scan followed by 30 MS/MS, each with a 40 ms accumulation time. First stage MS analysis was performed in positive ion mode, mass range m/z 400 − 1250 and 0.25 s accumulation time. Tandem mass spectra were acquired on precursor ions > 150 counts/s with charge state 2 − 5 and dynamic exclusion for 15 s with a 100 ppm mass tolerance. Spectra were acquired over the mass range of m/z 100 − 1500 using the manufacturer’s rolling collision energy based on the mass and charge of the precursor ion. The in-gel digests were analyzed using chromatographic separation (5 μL) employing a shorter linear gradient time frame from 3–25% solvent B over 38 min followed by: 5 min from 25% B to 33% B; 2 min 35% B to 80% B; 3 min at 80% B, 80–3% B, 1 min; and 8 min re-equilibration, and a wider mass range m/z 300–1250 for the data acquisition. Protein identification was undertaken using ProteinPilot™ 5.0.2 software (SCIEX) with searches conducted against the *Nicotiana benthamiana* of the Uniprot-SwissProt database (2018/08) appended with a custom nitrogenase database and a contaminant database (Common Repository of Adventitious Proteins). The total number of proteins in the custom database was 2622. The resulting proteomics data has been deposited to the CSIRO Data Access Portal (https://doi.org/10.25919/5f0d49f60d157).

#### Multiple reaction monitoring (MRM) method development

After database searching, tryptic peptides were selected for each of the four target proteins (AnfD, AnfK, AnfG, AnfH) (Supplementary Data Set 1). The selection of peptides was based on the criteria: MS response (high intensity), specific/unique to the target protein and of a size amenable to LC–MS (∼5–20 amino acids in length). The MS/MS data and subsequent database search results were used to select the highest intensity precursor ion (Q1 m/z) and product ions (Q3 m/z, n = 4) for each peptide, which were subsequently defined as the MRM method (Supplementary Table 1).

#### Targeted proteomics

Targeted liquid chromatography-multiple reaction monitoring-mass spectrometry (LC-MRM-MS) was performed for target protein detection. Reduced and alkylated tryptic peptides (15 μL) were chromatographically separated on a Kinetex C18 column (2.1 × 100 mm, Phenomenex) using a linear gradient of 5–45% acetonitrile in 0.1% formic acid, over 10 min at a flow rate of 400 μL/min. The eluate from the Shimadzu Nexera UHPLC was directed to a QTRAP 6500 mass spectrometer (SCIEX) equipped with a TurboV ion source operated in positive ion mode for data acquisition and analysis. The MS parameters were as follows: ion spray voltage, 5500 V; curtain gas, 35; GS1, 35; GS2, 40; source temperature, 500 °C; declustering potential, 70 V; and entrance potential, 10 V. Peptides were fragmented in the collision cell with nitrogen gas based on rolling collision energy dependent on the mass and charge of the precursor ion. Protein detection was confirmed using scheduled MRM scanning experiments with a 60 s detection window around the expected retention time (RT) and a 0.3 s cycle time. Data were acquired using Analyst v1.7 software. Peak areas of the four MRM transitions were integrated using Skyline (MacLean et al. 2010) wherein all transitions (n = 4) were required to co-elute with a signal-to-noise (S/N) > 3 and intensity > 1000 counts per second (cps) for detection (Supplementary Figs. 8–13). Two synthetic peptides (SpikeTides™ TQL; JPT Peptide Technologies GmbH, Berlin, Germany) were synthesised for AnfG and AnfH. These four peptides were isotopically labelled with stable isotopes at the C-terminal arginine (R; ^13^C6; ^15^N4) or lysine (K; ^13^C6; ^15^N2), resulting in a mass shift of + 10 or + 8 Da, respectively. The heavy peptides were used as standards to confirm the detection of the low abundant AnfG and AnfH light peptides (Supplementary Figs. 11 and 13).

#### Purification of proteins from plants under anaerobic conditions

*Nicotiana benthamiana* leaf samples were harvested four to five days after infiltration with *A. tumefaciens* containing the genetic construct of interest, snap-frozen in liquid nitrogen and stored in -80 until purification. Protein purifications were carried out in an anaerobic chamber (COY Laboratory Products) filled with a H_2_/N_2_ atmosphere (2–3%/97–98%). Anaerobic buffers were prepared at a Schlenk line in a bottle equipped with a butyl rubber septum by at least four cycles of evacuating and purging with N_2_. Leaf material of 10–20 g was macerated in 100 ml cold extraction buffer as above under anaerobic conditions (< 5 ppm O_2_) using a stick blender with 6 × five second pulses, keeping the mixture on ice throughout. The homogenised mixture was filtered through four layers of Miracloth and the filtrate (70–80 ml) centrifuged for 30 min at 20,000 x*g* at 4 °C. The supernatant was decanted and filtered through a 0.45 μM PVDF filter membrane to further remove fine particulates. The target protein was purified from the filtrate using two different methods as follows: In one method the filtrate (60–70 ml) was loaded onto a StreptactinXT column (2 mL bed volume, IBA Life Sciences) at 2 mL/min. The column was washed with 20 mL wash buffer, and protein containing the Twin-Strep epitope was eluted with elution buffer (50 mM biotin, 50 mM Tris, 75 mM NaCl, pH 8.0). In another method the filtrate was combined with 2 ml StreptactinXT resin (4 ml of 50% suspension, IBA Life Sciences) that was prewashed and resuspended in 5 ml wash buffer. The filtrate and resin mixture were gently mixed on a rotator wheel for 60 min and centrifuged for 5 min at 100 x*g*. The supernatant was removed to the surface of the pelleted resin, and resin with the remaining filtrate was transferred to a gravity flow column. The column was washed with 20 ml wash buffer and bound protein was eluted with elution buffer. The eluate was concentrated over a 10 kDa molecular weight cut-off membrane (10 kDa MWCO, Amersham) by centrifugation for 30 min at 3800 x*g*. The purified protein was frozen in liquid nitrogen for future analysis. Samples were retained from each step of the purification process for western blot analysis.

#### Affinity bead purification and imaging of plant mitochondria

*Agrobacterium tumefaciens* cells containing Twin-Strep-mTurquoise2-Metaxin (SN197) were infiltrated into *N. benthamiana* leaves, along with the p19 silencing suppressor and the multi-gene construct MTP-AnfDKGH (SL26). After four days, infiltration zones were harvested and the following steps were performed at 4 °C under standard laboratory atmospheric conditions. Leaf material was ground in 500 μL KPBS buffer (KPBS buffer: 1.36 M KCl, 100 mM KH_2_PO_4_.pH 7.25) using a mortar and pestle. The slurry was centrifuged at 1000 x*g* for 5 min. 300 μL of supernatant was applied to 50 μL of prewashed magnetic beads coated with streptavidin (DynaBeads MyOne C1, Thermo Fisher Scientific) in a 1.5 ml Eppendorf tube. The magnetic beads in the mixture were collected to the wall of the tube using a magnet and the remainder of the liquid was carefully removed. The magnetic beads were then washed twice with 1 mL KPBS buffer, and finally resuspended in 50 μL KPBS buffer. The purification process was analyzed via western blot. Streptavidin affinity beads post mitochondrial extraction were immediately mounted on a slide and imaged with a Leica SP8 laser-scanning confocal microscope (Leica Microsystems) equipped with a 40 × water-immersion objective (NA = 1.1) and operated via the LasX software. GFP (λ_ex_ = 488 nm; λ_em_ = 500–520 nm, gated at 0.30–3.80 ns) and transmitted light (λ_ex_ = 488 nm) were imaged in the same track.

## Results

### Expression and solubility of individual AnfDKGH proteins targeted to the plant mitochondrial matrix

**Fig. 1:**
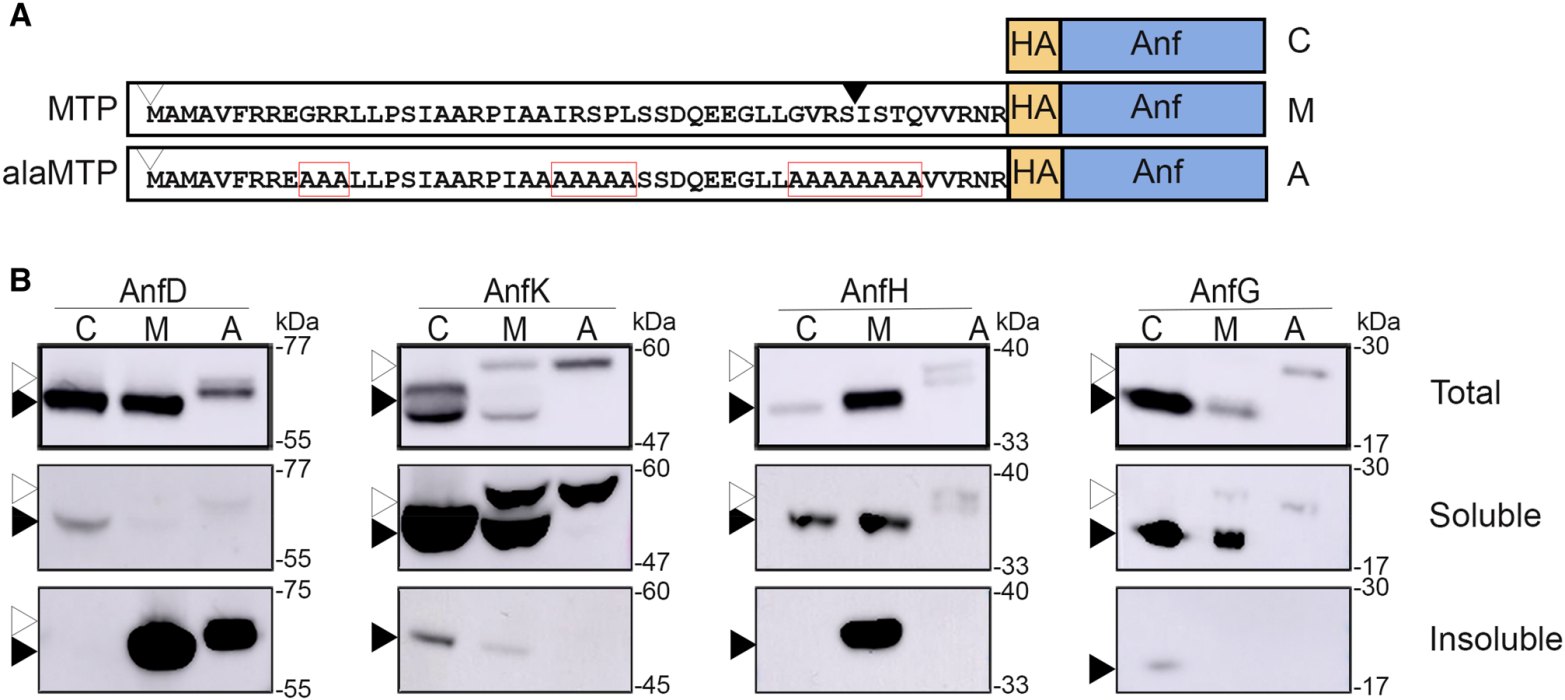
Abundance, processing, and solubility of individual Anf proteins when targeted to the plant cytosol or mitochondrial matrix. **A** Schematic representation of the Anf proteins and the amino acid sequence of the MTP and alaMTP, alanine substitutions highlighted by red boxes. **B** Upper panel, Total protein fraction of AnfD, AnfK, AnfH and AnfG; middle panel soluble fractions of AnfD, AnfK, AnfH and AnfG; lower panel, insoluble fractions of AnfD, AnfK, AnfH and AnfG. C, cytosolic expression; M, mitochondrially targeted; A, alaMTP. All western blots were probed with the α-HA antibody. Black arrowheads indicate the size of the MPP-cleaved proteins, white arrowhead the unprocessed protein. See Supplementary Fig. 1 for full blot and protein size ladders. See Table 1 for calculations of expected sizes for each Anf protein as full-length or processed proteins

As a first step towards expression of the Fe-nitrogenase within plants, we assessed the expression and localisation of AnfD, K, G and H to plant mitochondria using a transient *N. benthamiana* leaf-based expression system, as per our previous approaches to characterize the Nif proteins (Allen et al. 2017, 2020; Okada et al. 2020). These four *anf* genes from *Azotobacter vinelandii* were synthesised with codon optimization for dicotyledonous plant expression, and then further ‘domesticated’ for the Golden Gate cloning system to allow modular assemblies into various plant expression constructs (Table 1) (Peccoud et al. 2011). The mitochondrial targeting peptide (MTP) used predominantly in this study was a 51 amino acid (AA) peptide based upon the naturally occurring presequence of the *Arabidopsis thaliana* ATPase g subunit, pFAγ (Lee et al. 2012). This pFAγ51 MTP has been previously used for targeting Nif proteins to the matrix of plant mitochondria, and cleavage by the mitochondrial processing peptidase (MPP) results in a 9 AA extension at the N-terminus of the processed protein. For simplicity throughout this study, we refer to the 51 AA pFAγ51 targeting peptide as ‘MTP’ (Fig. 1A), and the 9 AA extension produced after MPP cleavage as the ‘scar’. As a control to detect unprocessed MTP-Anf proteins, we used a modified MTP with alanine substitutions to prevent cleavage within the mitochondrial matrix (Fig. 1A). Hereafter, this uncleavable form of the MTP is termed ‘alaMTP’ (Okada et al. 2020; Allen et al. 2020). To approximate the size of MPP-processed Anf proteins, we generated constructs that coded for Anf proteins that included a 10 AA N-terminal extension and were targeted to the cytosol; these controls include the prefix ‘cyto’ (Fig. 1A). The HA epitope was added to all Anf proteins for detection. The constructs were expressed transiently in *N. benthamiana* leaves using the 2 × 35S promoter from the *Cauliflower mosaic virus* (Kay et al. 1987), with a 5’ UTR enhancer element from the *Tobacco mosaic virus* (Gallie et al. 1987). Leaves were harvested four to five days post-infiltration, and total, soluble, and insoluble protein fractions were extracted and analyzed using SDS-PAGE and western blotting.

All versions of the AnfD, K, G and H proteins were detected in the total protein fractions, with the proteins migrating consistent with their predicted sizes (Fig. 1B, Table 1, Supplementary Fig. 1). Comparison of the migration patterns of the cyto-, alaMTP-, and MTP-Anf proteins, indicated that the four Anf proteins were targeted to the plant mitochondrial matrix and were processed by the MPP. Of particular note, the MTP-AnfD migrated as a single band at the expected size for correctly MPP-processed MTP. MTP-AnfK also appeared to be correctly processed by the MPP, however, the signal for scar-AnfK was partially obscured, possibly due to co-migration with the large subunit of RuBisCO (Ribulose-1,5-bisphosphate carboxylase) at ~ 50 kDa. MTP-AnfH and MTP-AnfG were detected predominantly at the size consistent with correctly MPP-processed proteins.

We next assessed the presence of AnfD, K, G and H in the soluble and insoluble protein fractions when targeted to either the cytosol, or to the mitochondrial matrix. This analysis revealed that the cytosolic and mitochondrial versions of AnfK and AnfG were found predominantly in the soluble protein fraction (Fig. 1B). Cytosolic AnfH was also found in the soluble protein fraction. However, mitochondrial-targeted AnfH was detectable in both the soluble and insoluble protein fractions, indicating that MTP-AnfH is only partially soluble in the plant mitochondria. We found that despite MTP-AnfD being correctly processed it was detected mostly in the insoluble fraction, in contrast to cytosolic AnfD which was soluble. Overall, the results indicated that when expressed individually, (i) AnfK and AnfG are correctly targeted, processed by the MPP and fully soluble, (ii) AnfH is correctly targeted and processed by the MPP but is only partially soluble, and (iii) AnfD was correctly targeted and processed by the MPP but was insoluble in the plant mitochondria.

### Coexpression of AnfDK within plant mitochondria improves AnfD solubility

**Fig. 2:**
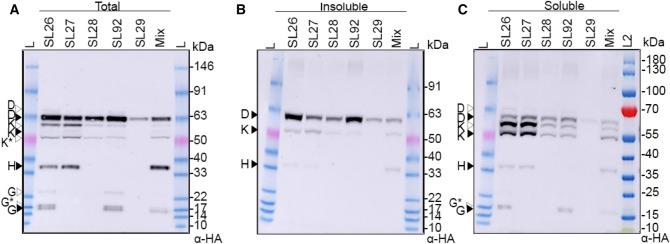
Abundance, processing and solubility of MTP-AnfD in the plant mitochondria when coexpressed with combinations of MTP-AnfK, MTP-AnfH and MTP-AnfG. Western blot analysis of multigene constructs with panels A, B and C showing the total, insoluble and soluble fractions respectively. Details of the constructs SL29, 27, 28, 29 and 92 can be found in Table 2. Note that Mix* represents a coinfiltration of Anf single gene constructs SN161 (*AnfD*), SN129 (*AnfK*), SN130 (*AnfH*) & SN131 (*AnfG*). L, L2-Ladders (L, Benchmark pre-stained protein ladder, L2, PageRuler pre-stained protein ladder). All western blots were probed with the α-HA antibody. Black arrow heads indicate the size of correctly processed Anf proteins, white arrowheads indicate the unprocessed proteins. K* indicates a faster migrating AnfK product, G* represents a ‘doublet’ band for AnfG. It should be noted that proteins migrated faster in the soluble and insoluble fractions due to the change in the composition of the solubility buffer

A series of multigene plant expression constructs were generated to coexpress AnfDK, AnfDKH, AnfDKG, and AnfDKGH within the plant mitochondria (Table 2). These constructs were expressed in *N. benthamiana* leaves and compared with expression of individual MTP-AnfD (SL29). Total, soluble, and insoluble protein fractions were analyzed via SDS-PAGE and western blotting, using a HA antibody (Fig. 2). It should be noted that proteins migrated slightly faster in the soluble and insoluble fractions due to slight changes in the osmolarity of the solubility buffer. All Anf proteins coded in each multigene construct were detected by western blot, with the following observations (i) encouragingly, coexpression of AnfK with AnfD improved the expression of AnfD in the soluble fraction (compare lanes SL29 to SL28 in Fig. 2C); (ii) coexpression of AnfD, AnfK and AnfH (SL27) increased abundance of AnfK in the soluble fraction (Fig. 2C); and (iii) coexpression of AnfD, AnfK and AnfG (SL92) resulted in signals for MTP-AnfG and scar-AnfG, as well as another slightly larger product at a size just above the scar-AnfG protein band (~ 17 kDa). These two signals for AnfG are potentially a staggered MPP-cleaved scar-AnfG (Fig. 2A, B, C). We also compared coexpression of AnfD, AnfK, AnfG and AnfH from a single multigene construct (SL26), to coexpression from four individual constructs at a 1:1:1:1 *Agrobacterium* culture density, (lane labelled Mix, Fig. 2A). This comparison demonstrated that although mixing individual constructs produced signals for AnfDKGH, the abundance of the AnfD, AnfK and AnfG was slightly higher when using a single construct containing all four genes.

### AnfDKGH is correctly processed when targeted to plant mitochondria

**Fig. 3:**
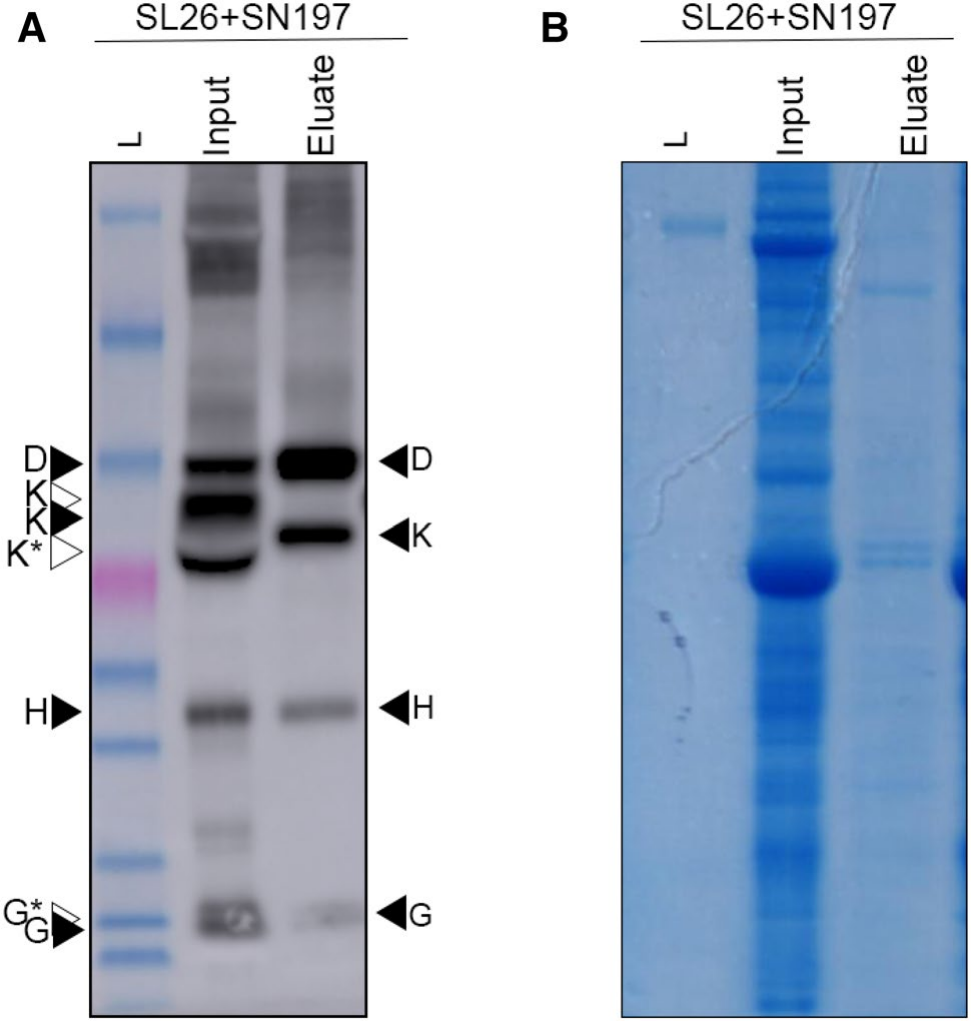
Western blot analysis of isolated plant mitochondria coexpressing MTP-AnfD, MTP-AnfK, MTP-AnfH and MTP-AnfG **A** Western blot (α-HA) comparison of SL26 (AnfD, K, H and G, Table 2) before and after isolation of plant mitochondria coexpressing SN197. Brightness and contrast have been adjusted for this image. **B** Post-transfer Coomassie stained SDS-PAGE gel comparing SL26 (AnfD, K, H and G, Table 2) before and after isolation of plant mitochondria coexpressing SN197. Black arrow heads indicate the size of correctly processed Anf proteins, white arrowheads indicate the unprocessed proteins. K* indicates a faster migrating AnfK product. G* represents a ‘doublet’ band for AnfG. For ladder sizes refer to Fig. 2

To further investigate the mitochondrial import and subsequent MPP cleavage of the Anf proteins we performed a mitochondrial pulldown using a construct coding for Twin-Strep-mTurquoise-metaxin (SN197) (Allen et al. 2020) (Supplementary Fig. 2). Preliminary experiments using streptavidin-coated magnetic beads indicated that coexpression of SN197 and MTP-GFP in *N. benthamiana* permitted the rapid isolation of GFP-labelled plant mitochondria (Supplementary Fig. 2). Next, SN197 and MTP-AnfDKGH (SL26, Table 2) were coinfiltrated into *N. benthamiana* leaves. After four days, leaf material was harvested and mitochondria isolated. Comparison of the protein extracts before and after mitochondrial isolation indicated the presence of MPP-processed AnfD, AnfK, AnfG and AnfH, within plant mitochondria (Fig. 3A). Interestingly, correctly processed AnfK was detectable in the purified 'eluate' sample, despite not being observed in the ‘input’ sample. This is likely due to the removal of the abundant RuBisCO protein that masks the signal in unpurified samples. MPP-processed AnfG was less abundant than AnfD, AnfK and AnfH. Finally, LC–MS/MS based proteomics was conducted on excised AnfD, K, G and H proteins from the Coomassie stained gel (Fig. 3B, Supplementary Data Set 2). This analysis confidently identified AnfD, AnfK and AnfH, but not AnfG (Supplementary Data Set 2).

### AnfD and AnfK form a protein complex in the plant mitochondrial matrix

**Fig. 4:**
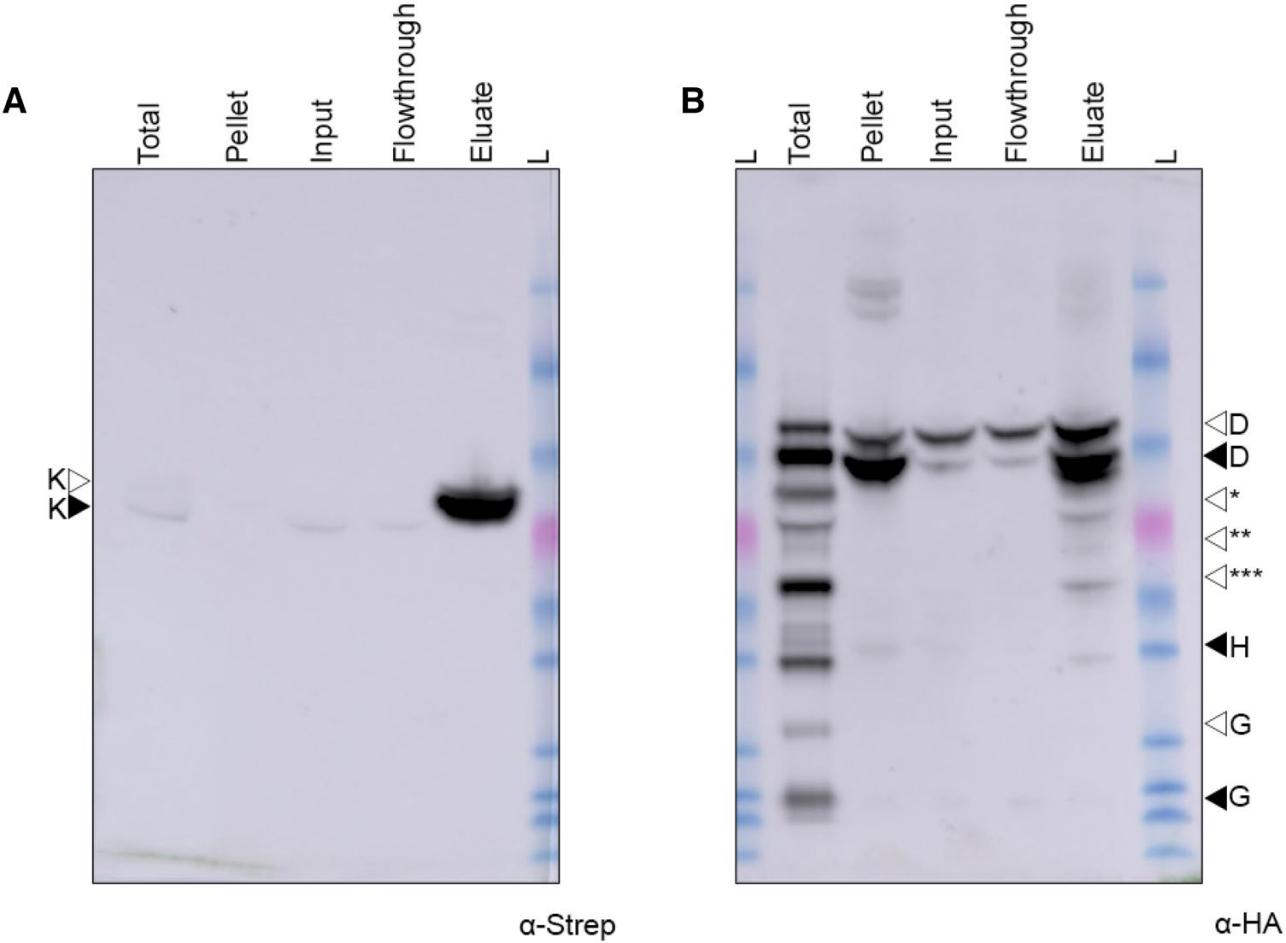
Purification of Twin-Strep-AnfK from plant mitochondria copurifies AnfD **A** Western blot (α-Strep) analysis of various stages taken during the purification process of Twin-Strep-tagged MTP-AnfK. **B** Western blot (α-HA) analysis of various stages taken during the purification process of Twin-Strep-tagged MTP-AnfK. Black arrow heads indicate the size of correctly processed Anf proteins, white arrowheads indicate the unprocessed proteins. *, **, *** indicates potential degradation products. For ladder sizes refer to Fig. 2

The strong interaction of AnfD and AnfK to form the catalytic dinitrogenase in diazotrophs (Chisnell et al. 1988) is critical to realise function in plants. We investigated this interaction in plant mitochondria using StrepTactin™-based affinity chromatography. AnfK was translationally fused at the N terminus to the CoxIV mitochondrial targeting peptide (CoxIV), followed by a Twin-Strep tag (Lee et al. 2012; Buren et al. 2017), generating a final construct named CoxIV-Twin-Strep-AnfK. AnfD, AnfG and AnfH were fused with the MTP and HA epitope, assembled along with CoxIV-Twin-Strep-AnfK into a multigene expression construct (SL37, Table 2) and infiltrated into *N. benthamiana* leaves. Five days after infiltration Twin-Strep-AnfK was purified under anaerobic conditions. The different stages of the purification were analyzed using SDS-PAGE and probed via western blot using either StrepTactin™ or HA antibodies (Fig. 4A, B). As expected, Twin-Strep-AnfK was readily purified and detected in the eluate, migrating through the gel at a speed consistent with a protein processed by the MPP (Fig. 4A). Notably, western blot analysis of the eluate using the HA antibody showed that AnfD was co-purified with AnfK. However, the ratio of AnfD to AnfK was lower than 1:1 likely due to the lower abundance of AnfD in the soluble fraction compared to AnfK (Supp Fig. 4). Two bands were detected for AnfD, corresponding to full-length, unprocessed MTP-AnfD, and the MPP-processed AnfD (Fig. 4B). An additional band was also detected directly below the size expected for processed AnfD, which migrated slightly faster than the AnfD signal as a doublet. This area around the doublet of AnfD was excised from a protein gel and confirmed via proteomics to include AnfD peptides. The eluate was also analyzed using discovery proteomics to additionally confirm the presence of AnfK and AnfD proteins, ranked number 1 and 2, respectively, of 520 identified proteins (Supplementary Figs. 3 & 4, Supplementary Data set 4). LC-MRM-MS also confirmed the presence of both AnfH and AnfG with low but detectable response (Supplementary Figs. 6–11). Western blotting also revealed faint bands corresponding to the sizes expected for scar-AnfH and AnfG proteins within the eluate sample (Fig. 4B), consistent with their detection by proteomics. These results indicate that AnfD and AnfK interact with one another when expressed within the plant mitochondrial matrix, and AnfH and AnfG interact weakly with AnfDK.

### AnfD and AnfK copurify with AnfG when expressed in the plant mitochondrial matrix

**Fig. 5:**
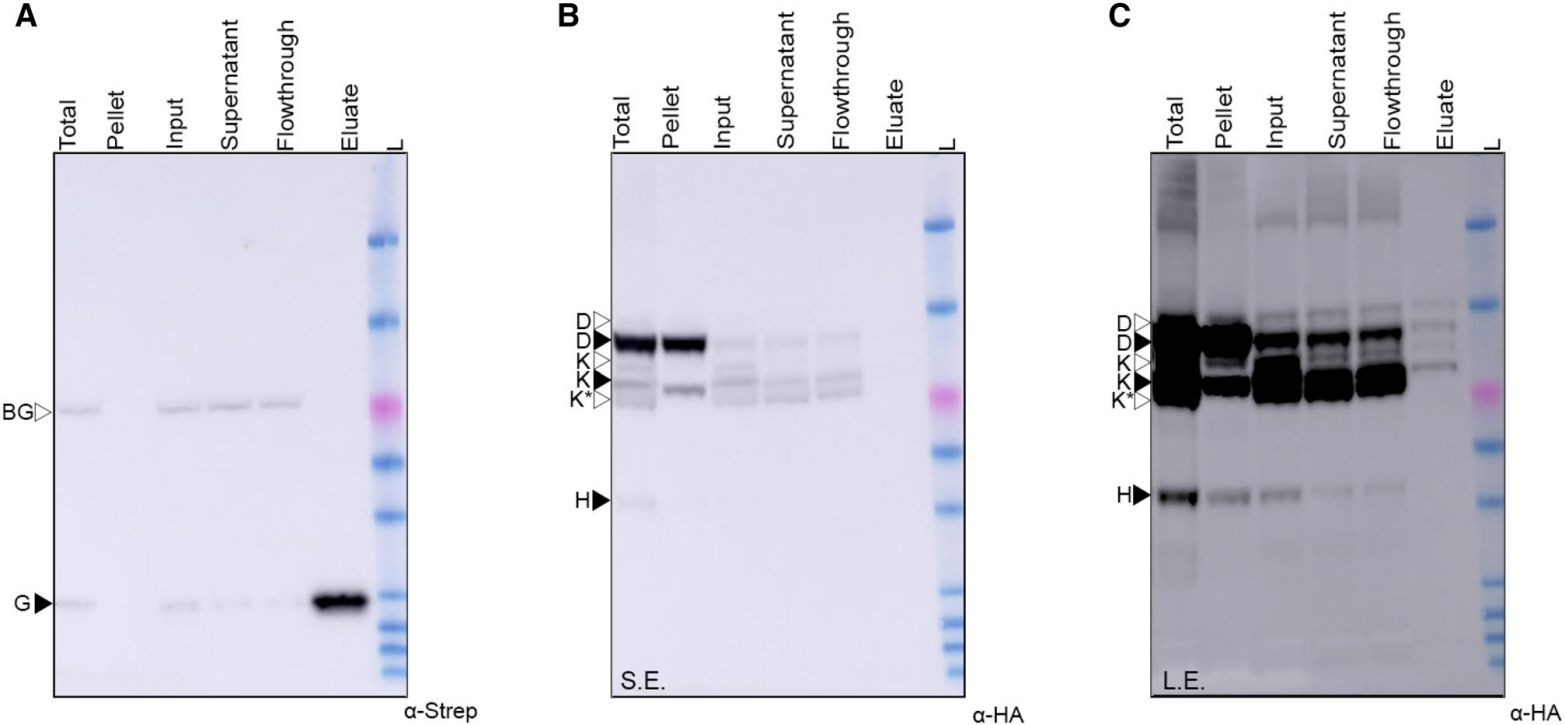
Purification of Twin-Strep-AnfG from plant mitochondria copurifies both MTP-AnfD and MTP-AnfK **A** Western blot (α-Strep) analysis of various stages taken during the purification process of Twin-Strep-tagged MTP-AnfG, 10 s exposure. **B** Western blot (α-HA) analysis of various stages taken during the purification process of Twin-Strep-tagged MTP-AnfG, short 2 min exposure, (S.E.). **C** Western blot (α-HA) analysis of various stages taken during the purification process of Strep-tagged MTP-AnfG, long 20 min exposure (L.E.). Black arrow heads indicate the size of correctly processed Anf proteins, white arrowheads indicate the unprocessed proteins. K* indicates a faster migrating product of AnfK. BG indicates background band. For ladder sizes refer to Fig. 2.

To further assess the interaction of AnfG with AnfDKH, we attempted to pull down AnfDK using Twin-Strep tagged AnfG as bait. The other AnfDKH proteins were translationally fused with MTP and HA epitope on the N-terminus (SL93, Table 2). SL93 was infiltrated into *N. benthamiana* leaves, Twin-Strep-AnfG was purified under anaerobic conditions and analysed by SDS-PAGE and western blot (Fig. 5). Twin-Strep-AnfG was detected in the eluate, migrating as a correctly processed mitochondrial matrix-targeted protein (Fig. 5A). Western blot analysis of the eluate with the HA antibody revealed a weak detection of both MPP-processed, and unprocessed versions of AnfD and AnfK (Fig. 5B, C). Bands corresponding to AnfD and AnfK were only visible after a 20 min exposure, with these weak signals contrasting with the strong AnfG signal visible after a short 10 s exposure. Although AnfH was detected in the total protein sample, it was not detected within the eluate even after the longer 20 min exposure time. The eluate was also analyzed using discovery proteomics to additionally confirm the presence of AnfG, AnfD, and AnfK proteins identified as number 1, 11, and 44, respectively, in the list of 127 identified proteins (Supplementary Data set 5). LC-MRM-MS confirmed the presence of AnfG, AnfD, and AnfK in the eluate sample as well as confirming the absence of AnfH (Supplementary Data set 6). Taken together, western blot and proteomic analysis indicate that AnfG weakly interacts with AnfDK within the matrix of plant mitochondria.

## Discussion

A prerequisite for a functional Fe-nitrogenase in plant mitochondria is that Anf proteins need to be successfully targeted, correctly folded and interact with each other. Here we expressed AnfD, K, G and H in plant mitochondria and demonstrated that AnfD and AnfK form a complex. This study provides the foundation for engineering the Fe-nitrogenase into plants.

We found that AnfD was insoluble when expressed in the absence of AnfK in plant mitochondria. However, coexpression of AnfD with AnfK enabled a protein–protein interaction with AnfK and enhanced the solubility of AnfD. This suggests that AnfD and AnfK are correctly folded when coexpressed in the organelle. In bacteria the structure of the heterotetrameric complex of the Fe-nitrogenase stabilises AnfDK (Chisnell et al. 1988; Trncik et al. 2023). Given this, our observed interaction of AnfD and AnfK is not necessarily surprising, although it remains encouraging that this can be recapitulated in plant mitochondria. This result reflects similar findings for *K. oxytoca* NifD, which was also insoluble when targeted to the plant mitochondrial matrix, but its solubility was improved when linked to NifK as a translationally fused polyprotein, NifD-linker-NifK (Allen et al. 2020). Interestingly, AnfD was soluble when expressed individually in the plant cytosol. This example of contrasting solubilities for the same protein but located in different compartments of a cell could be related to differences in pH, chaperones, and temperature, and highlights the need for more research into the basis of protein folding.

In contrast to the strong interaction observed for AnfD and AnfK we found a weak interaction between AnfG and AnfDK. This result in plants is different to that reported in *A. vinelandii* and in yeast mitochondria (Lopez-Torrejon et al. 2021; Pérez-González et al. 2021). In *A. vinelandii* the insertion of the FeFe-cofactor into AnfDK is considered a pre-requisite for stable binding of the AnfG to the AnfDK complex. In the absence of NifB, the biogenesis of the FeFe-cofactor is impeded, and AnfG is not found to bind stably to AnfDK (Pérez-González et al. 2021). Similarly, In yeast co-expression of AnfDKG with AnfH resulted in the purification of AnfDK without detectable levels of AnfG (Lopez-Torrejon et al. 2021). In our plant-based experiments the complex of AnfDKG does not contain any active FeFe-cofactor, as NifB and other components required for cofactor biogenesis were not included. In the absence of the FeFe-cofactor our results may reflect that the interaction of AnfG with AnfDK is in a hexameric form that is unstable. These contrasting results may reflect differences in purification, analysis methods and host expression systems, and the relevance of this interaction in the absence of the FeFe-cofactor remains to be determined.

Similarly, AnfH was weakly detected when AnfK was purified from plant mitochondria, and not detected in the purification of AnfG. This potentially indicates that AnfH is interacting with either AnfK or AnfD but not with AnfG. This is in accordance with the predicted transient interaction of AnfH with the AnfDKG complex, analogous to the transient interaction of NifH with NifDK (Burgess and Lowe 1996). Although the interactions shown here are somewhat reflective of the native bacterial environment, it is also possible that the addition of the N-terminal HA and Twin-Strep epitope tags could impact the protein–protein interactions between AnfH, D, K, and G. Likewise, the use of MTPs in this study has the potential to impact protein–protein interactions due to the ‘scar’ amino acid residues left after cleavage of the N-terminal MTP. Future studies would likely focus on mitochondrial expression of the Anf proteins without the use of epitope tags, and to identify MTPs that will leave minimal or no ‘scar’ residue.

In comparison to the lack of soluble *A. vinelandii* NifH when expressed in plant mitochondria (Jiang et al. 2021) we found that AnfH was partially soluble. However, unlike AnfD, AnfH solubility was not significantly improved by coexpression with AnfDKG. Encouragingly, AnfH expressed in yeast mitochondria was soluble and active in vitro for donation of electrons to NifDK (Lopez-Torrejon et al. 2021). What underlies this difference in AnfH solubility between the two organisms remains to be determined. As AnfH is a key structural component, future experiments will need to focus on improving the solubility of AnfH in plant mitochondria.

As we have previously observed for Nif proteins expressed in plant mitochondria (Allen et al. 2017; Okada et al. 2020), expression and processing efficiency of the Anf proteins varied despite use of the same promoter and MTP. Furthermore, these differences were consistent regardless of whether the Anf proteins were expressed individually, or together via multigene constructs. Further modulation and the use of alternate MTPs could conceivably improve both abundance and processing efficiency.

In its native bacterial host, a large proportion of protein content is comprised of nitrogenase proteins when under diazotrophic growth (Waite et al. 2021). However, it is unknown what levels of AnfH, D, K, and G will be required to produce sustainable nitrogen fixation in plants when grown in their terrestrial environment. In this study we did not determine the overall abundance of the Anf proteins, although in addition to the issues of nitrogenase assembly, future studies could focus on determination of the protein content and expression levels that are required in the plant mitochondrial matrix for nitrogen fixation.

Despite showing a partial reconstruction of the Fe-nitrogenase within plant mitochondria additional Nif proteins will be required for an active Fe- nitrogenase. Currently *nifF, nifJ, nifS, nifU, nifV* and *nifB* as well as *anfD, anfK, anfG* and *anfH* are considered the minimal gene set required for Fe- nitrogenase activity in *Escherichia coli* (Yang et al. 2014). It is likely all these proteins will need to be expressed in plant mitochondria, as endogenous plant equivalents either do not exist or cannot functionally substitute (Yang et al. 2017). Furthermore, the assembly and stability of the nitrogenase iron-sulphur clusters will need to be investigated as recent studies have shown incomplete loading of [FeS] clusters on Nif proteins when isolated from plant mitochondria (Jiang et al. 2021). Nevertheless, the initial steps towards plant engineering of a Fe-nitrogenase have been demonstrated here, and encouragingly the catalytic AnfDK subunit has been shown to form a complex within mitochondria.

## Supplementary Information

Below is the link to the electronic supplementary material.

Supplementary file1 (XLSX 76 KB)

Supplementary file2 (DOCX 15591 KB)

## Data Availability

Proteomics data has been deposited to the CSIRO Data Access Portal (https://doi.org/10.25919/5f0d49f60d157).
