# Supplementary material for: The structural components of the Azotobacter vinelandii iron-only nitrogenase, AnfDKG, form a protein complex within the plant mitochondrial matrix
Source: Plant Mol Biol. 2023 Jun 16;112(4-5):279–91. doi: 10.1007/s11103-023-01363-3 (PMC10352409; doi:10.1007/s11103-023-01363-3)
Supplement: Supplementary file 2 — Supplementary file2 (DOCX 15591 KB) [file 11103_2023_1363_MOESM2_ESM.docx]

**Supplementary material**

**The structural components of the *Azotobacter vinelandii* iron-only nitrogenase, AnfDKG, form a protein complex within the plant mitochondrial matrix**

Johnston E.^1,5^, Okada S.^1^, Gregg C. M.^2^, Warden A. C.^1^, Rolland V.^2^, Gillespie V.^2^, Byrne K.^3^, Colgrave M. L.^3,4^, Eamens A. L.^6^, Allen R. S.^2*^, Wood C. C.^2^

^1^CSIRO Environment, GPO Box 1700, Acton, ACT 2601, Australia

^2^CSIRO Agriculture and Food, GPO Box 1700, Acton, ACT 2601, Australia

^3^CSIRO Agriculture and Food, 306 Carmody Rd, St Lucia, QLD 4067, Australia

^4^Australian Research Council Centre of Excellence for Innovations in Peptide and Protein Science, 306 Carmody Rd, St. Lucia, QLD 4067, Australia

^5^School of Environmental and Life Sciences, University of Newcastle, University Dr, Callaghan NSW 2308, Australia

^6^School of Health, University of the Sunshine Coast, Maroochydore, QLD 4558, Australia

*corresponding author rob.allen@csiro.au

Table of Contents

[**Supplementary Figure 1|** Analysis of total, soluble and insoluble protein fractions of individual Anf proteins when targeted to the plant cytosol or mitochondrial matrix. 4](#_Toc133155126)

[**Supplementary Figure 2|** Mitochondrial enrichment by affinity purification 5](#_Toc133155127)

**Supplementary Figure 3|** Coomassie stained gel, post PVDF membrane transfer of abundance, processing and solubility of MTP-AnfD in the plant mitochondria when coexpressed with combinations of MTP-AnfK, MTP-AnfH and MTP-AnfG……………………………………………………………………..6

**Supplementary Figure 4|** SDS-PAGE analysis of various stages taken during the purification process of Twin-Strep-tagged MTP-AnfK………………………………………………………………………………………………………7

**Supplementary Figure 5|** SDS-PAGE analysis post *PVDF membrane* transfer of various stages taken during the purification process of Twin-Strep-tagged MTP-AnfG………………………………………………………8

**[Supplementary Figure 6|](#_Toc133155128)** [Extracted ion chromatogram (XIC) for AnfD tryptic peptides from the eluate of the StrepTactin purification from leaves infiltrated with SL37. 9](#_Toc133155128)

**[Supplementary Figure 7|](#_Toc133155129)** [Extracted ion chromatogram (XIC) for AnfK tryptic peptides from the eluate of the streptactin purification from leaves infiltrated with SL37. 10](#_Toc133155129)

**[Supplementary Figure 8|](#_Toc133155130)** [Extracted ion chromatogram (XIC) for AnfH tryptic peptides from the eluate of the streptactin purification from leaves infiltrated with SL37. 11](#_Toc133155130)

**[Supplementary Figure 9|](#_Toc133155131)** [Extracted ion chromatograms (XIC) for four selected peptides (I) ITNDDVIK, (II) EFLEEFTAAIGTK, (III) EYGELAR and (IV) DNIVQK using MRM for AnfH. 12](#_Toc133155131)

**[Supplementary Figure 10|](#_Toc133155132)** [Extracted ion chromatogram (XIC) for AnfG tryptic peptides from the eluate of the StrepTactin purification from leaves infiltrated with SL37. 13](#_Toc133155132)

**[Supplementary Figure 11|](#_Toc133155133)** [Extracted ion chromatograms (XIC) for three selected peptides; (I) IDELTDYIMK, (II) ELLCGEPVDLSTSHDR and (III) EEIGSLMQGLK using MRM for AnfG. 14](#_Toc133155133)

**[Supplementary Table 1|](#_Toc133155134)** [Multiple reaction monitoring transitions of AnfG, H, D and K peptides for targeted liquid chromatography – mass spectrometry (LC-MS). 15](#_Toc133155134)

**[Supplementary Data Set 1|](#_Toc133155135)** [Schematic outline of the peptides detected by LC-MS/MS for AnfDKGH isolated from a range of plant-based expression experiments 21](#_Toc133155135)

**[Supplementary Data Set 2|](#_Toc133155136)** [Schematic outline of the peptides detected by LC-MS/MS for MTP-AnfDKH from enriched plant mitochondria. 22](#_Toc133155136)

**[Supplementary Data Set 3|](#_Toc133155137)**[Codon-optimized DNA sequences and translated protein sequences of](#_Toc133155137) *[anfD](#_Toc133155137)*[,](#_Toc133155137) *[anfK](#_Toc133155137)*[,](#_Toc133155137) *[anfG](#_Toc133155137)* [and](#_Toc133155137) *[anfH](#_Toc133155137)* [genes used in this study. 24](#_Toc133155137)


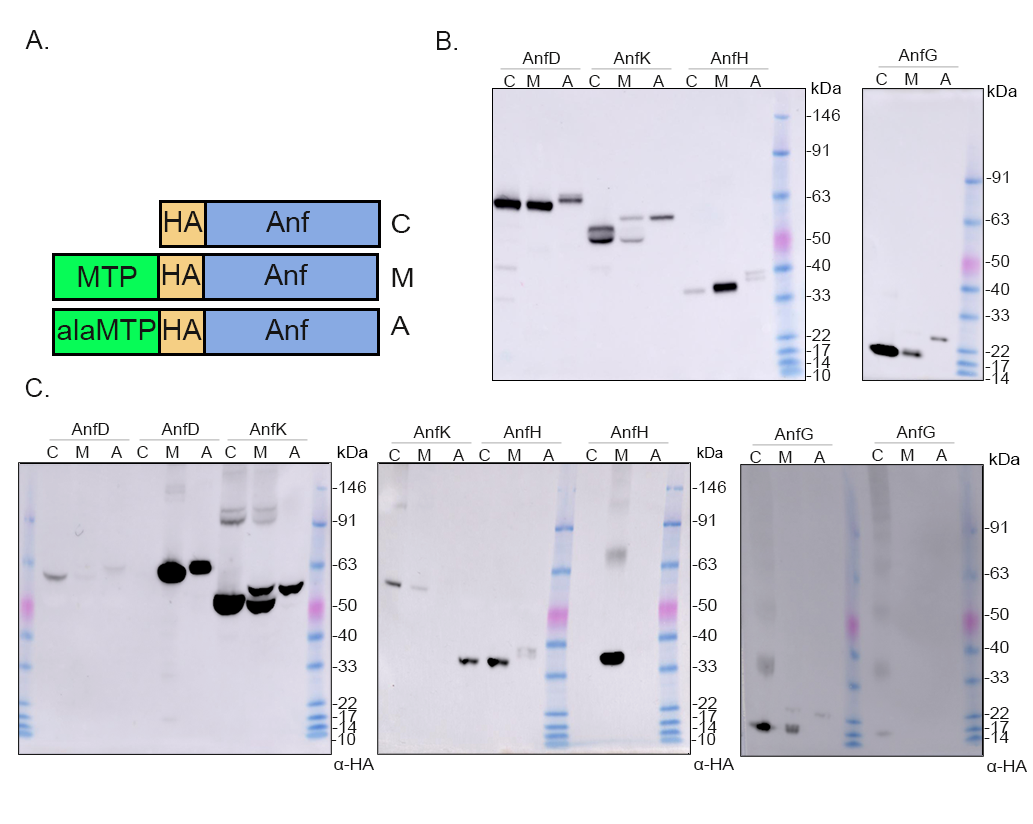


### **Supplementary Figure 1|** Analysis of total, soluble and insoluble protein fractions of individual Anf proteins when targeted to the plant cytosol or mitochondrial matrix.

**A)** Schematic representation of the MTP-HA-Anf, alaMTP-HA-Anf and the HA-Anf constructs. **B)** Western blot analysis of AnfD, AnfK, AnfH and AnfG in total protein extracts from *N. benthamiana* leaf. **C)** Western blot analysis of AnfD, AnfK, AnfH and AnfG in soluble and insoluble protein extracts from *N. benthamiana* leaf. C, cytosolic expression; M, mitochondrial targeted; A, alanine scanned MTP. Parts of these western blot images were used to construct Fig. 1 in the main text.

**
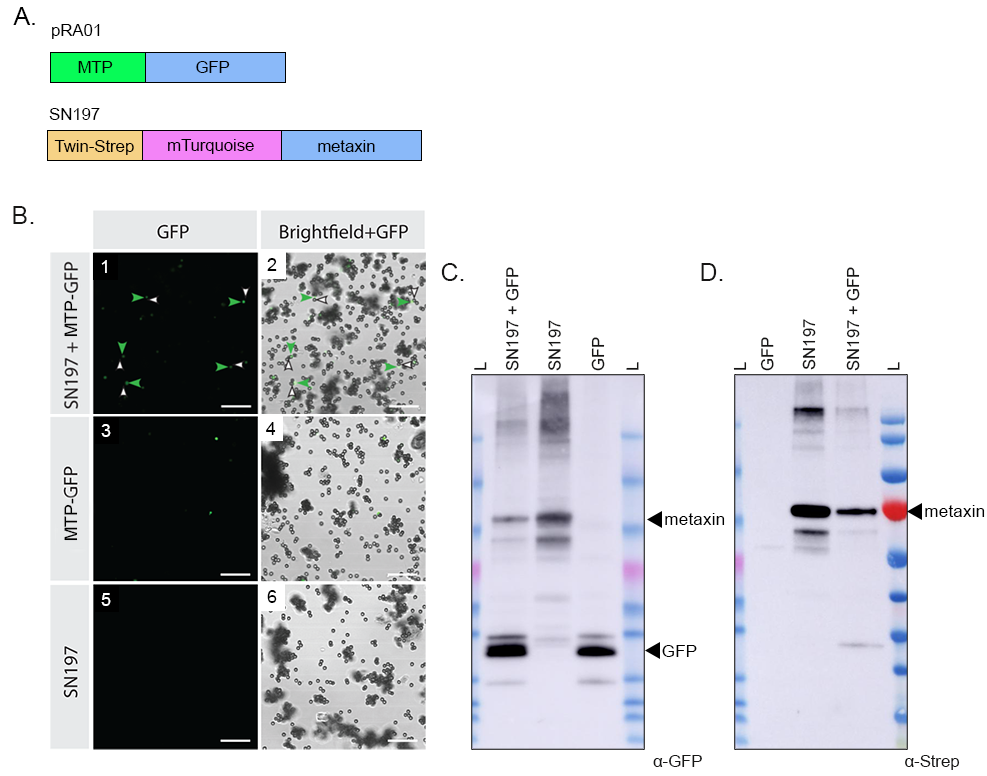
**

### **Supplementary Figure** **2|** Mitochondrial enrichment by affinity purification

1. Schematic representation of constructs MTP-GFP, pRA01, and Twin-Strep-mTurquoise-metaxin, SN197. **B)** Images of mitochondrial enrichment by affinity purification using Twin-Strep-mTurquoise-metaxin. Panel 1 and 2 show the enrichment of fluorescent mitochondria from leaves expressing both, MTP-GFP and Twin-Strep-mTurquoise-metaxin. Panel 3 and 4 show the eluate of the negative control sample, from leaves expressing MTP-GFP only. Panel 5 and 6 show the eluate from leaves expressing Twin-Strep-mTurquoise-metaxin only. The imaging revealed an increase in GFP florescence for the sample expressing both MTP-GFP and Twin-Strep-mTurquoise-metaxin compared to either of the controls. Panels 1, 3, 5 show the fluorescent channel, dark field image. Panels 2, 4 and 6 show the fluorescent channel, bright field image. The images shown are representative of three biological replicates. **C)** Western blot (α-GFP) analysis of eluate samples from the mitochondrial enrichment. **D)** Western blot (α-Strep) analysis of eluate samples from the mitochondrial enrichment. Western blots were probed with the α-GFP antibody for GFP and α-strep antibody for visualization of metaxin. Twin-Strep-mTurquoise-metaxin was also detected as indicated by α-GFP due to the sequence similarity of mTurquoise and GFP.


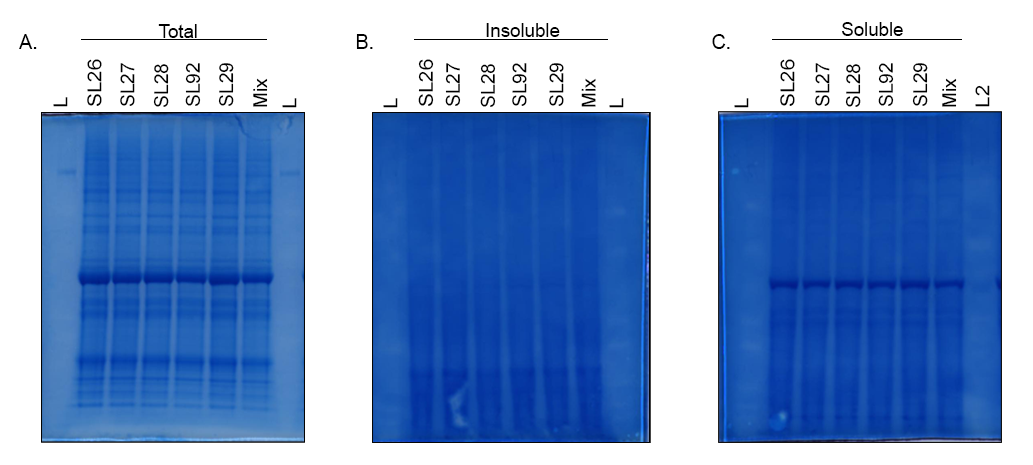


**Supplementary Figure 3|** Coomassie stained gel, post PVDF membrane transfer of abundance, processing and solubility of MTP-AnfD in the plant mitochondria when coexpressed with combinations of MTP-AnfK, MTP-AnfH and MTP-AnfG.

Panels A, B and C show the Coomassie stained SDS-PAGE total, insoluble and soluble fractions respectively. Details of the constructs SL29, 27, 28, 29 and 92 can be found in Table 2. Corresponding western blot figures in main text, Figure 2. Note that Mix* represents a coinfiltration of Anf single gene constructs SN161 (AnfD), SN129 (AnfK), SN130 (AnfH) & SN131 (AnfG). L, L2-Ladders (L, Benchmark pre-stained protein ladder, L2, PageRuler pre-stained protein ladder).


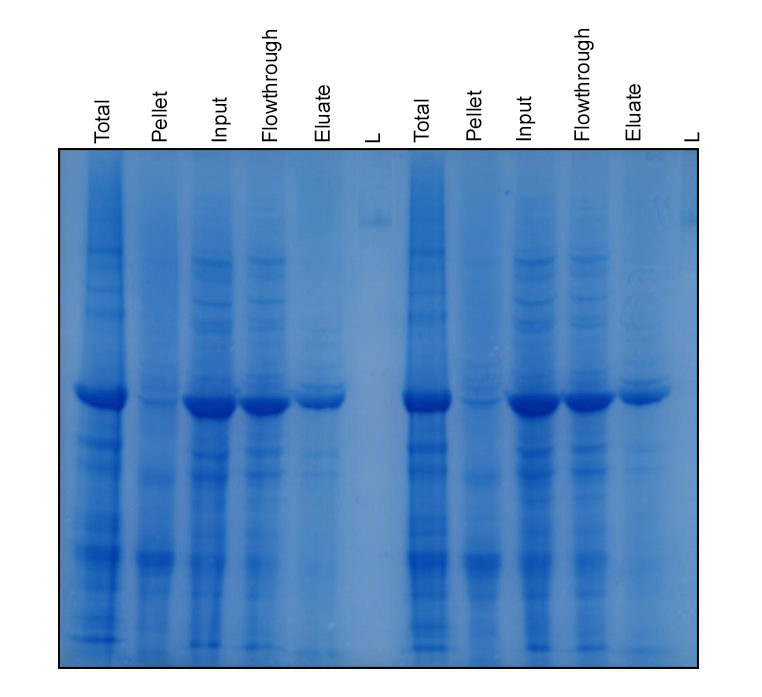


**Supplementary Figure 4|** SDS-PAGE analysis of various stages taken during the purification process of Twin-Strep-tagged MTP-AnfK. Corresponding western blot figures in main text, Figure 4. L, L2-Ladders (L, Benchmark pre-stained protein ladder, L2, PageRuler pre-stained protein ladder).


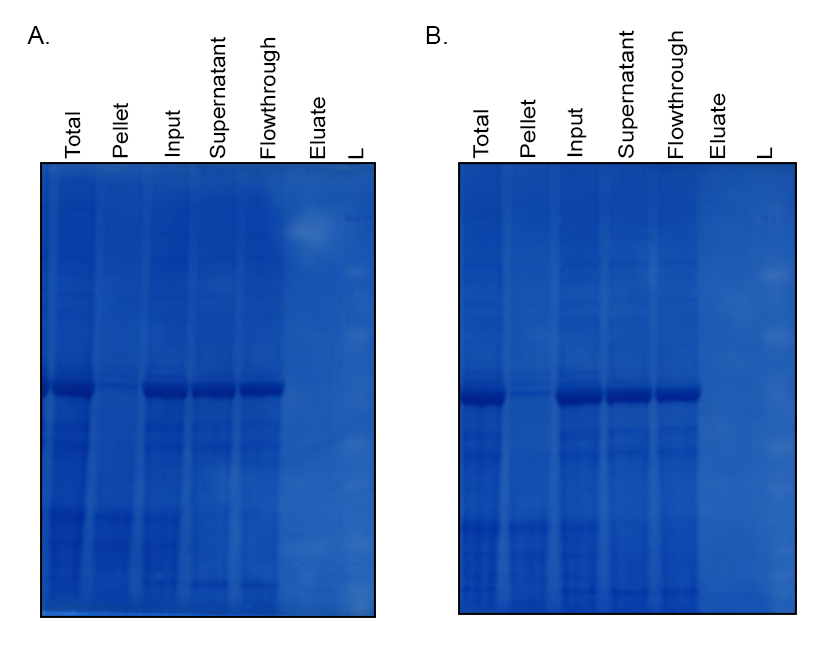


**Supplementary Figure 5|** SDS-PAGE analysis post PVDF membrane transfer of various stages taken during the purification process of Twin-Strep-tagged MTP-AnfG. Small proteins are likely not visible due to transfer to PVDF membrane for western blot analysis. Corresponding western blot figures in main text, Figure 5. L, L2-Ladders (L, Benchmark pre-stained protein ladder, L2, PageRuler pre-stained protein ladder).

**AnfD**

**
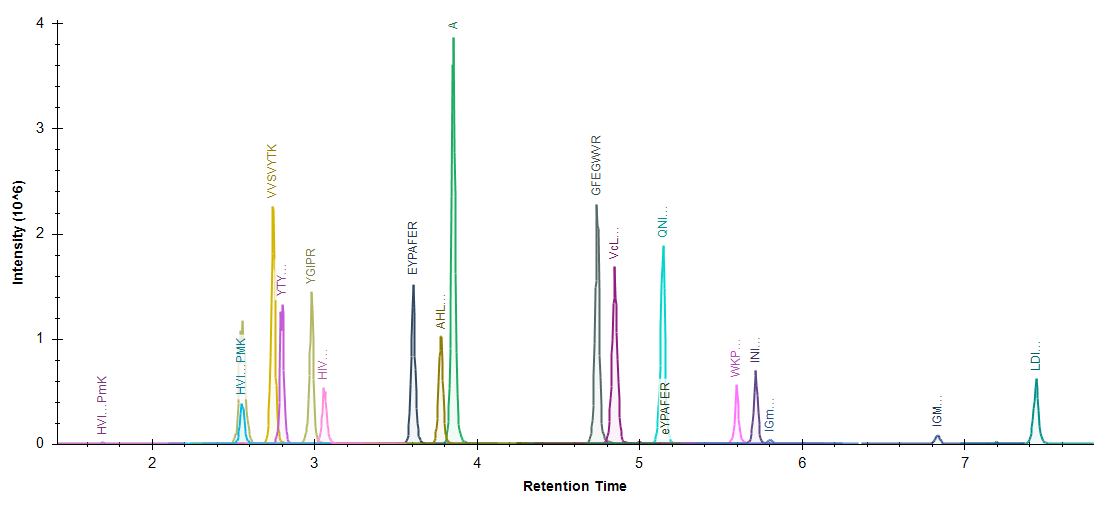
**

### **Supplementary Figure 6|** Extracted ion chromatogram (XIC) for AnfD tryptic peptides from the eluate of the StrepTactin purification from leaves infiltrated with SL37.

Fourteen MRM peptides were assessed for AnfD. Each peptide displayed peak profiles with high response and the product ions (Q3 m/z, n = 4) profiled with the same retention times.

**AnfK**

**
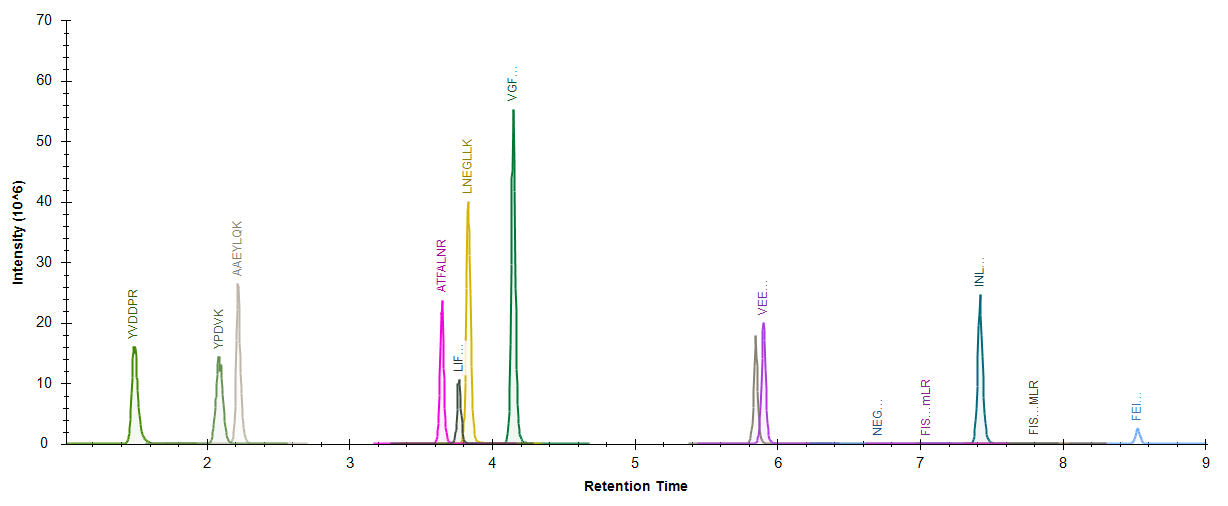
**

### **Supplementary Figure 7|** Extracted ion chromatogram (XIC) for AnfK tryptic peptides from the eluate of the streptactin purification from leaves infiltrated with SL37.

Thirteen MRM peptides were assessed for AnfK. Each peptide displayed peak profiles with high response and the product ions (Q3 m/z, n = 4) profiled with the same retention times.

**AnfH**


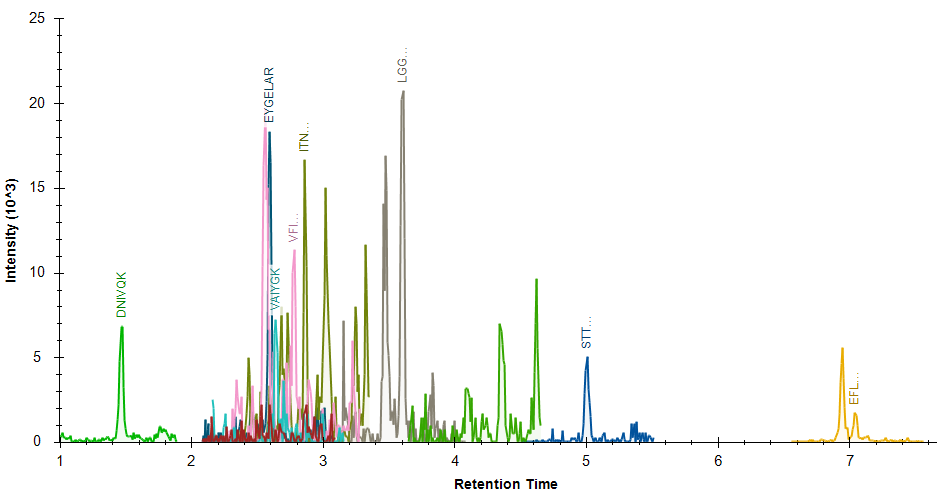


### **Supplementary Figure 8|** Extracted ion chromatogram (XIC) for AnfH tryptic peptides from the eluate of the streptactin purification from leaves infiltrated with SL37.

Eight MRM peptides were assessed for AnfH. Each peptide displayed peak profiles with low but detectable response and the product ions (Q3 m/z, n = 4) profiled with the same retention times.

**AnfH**

**
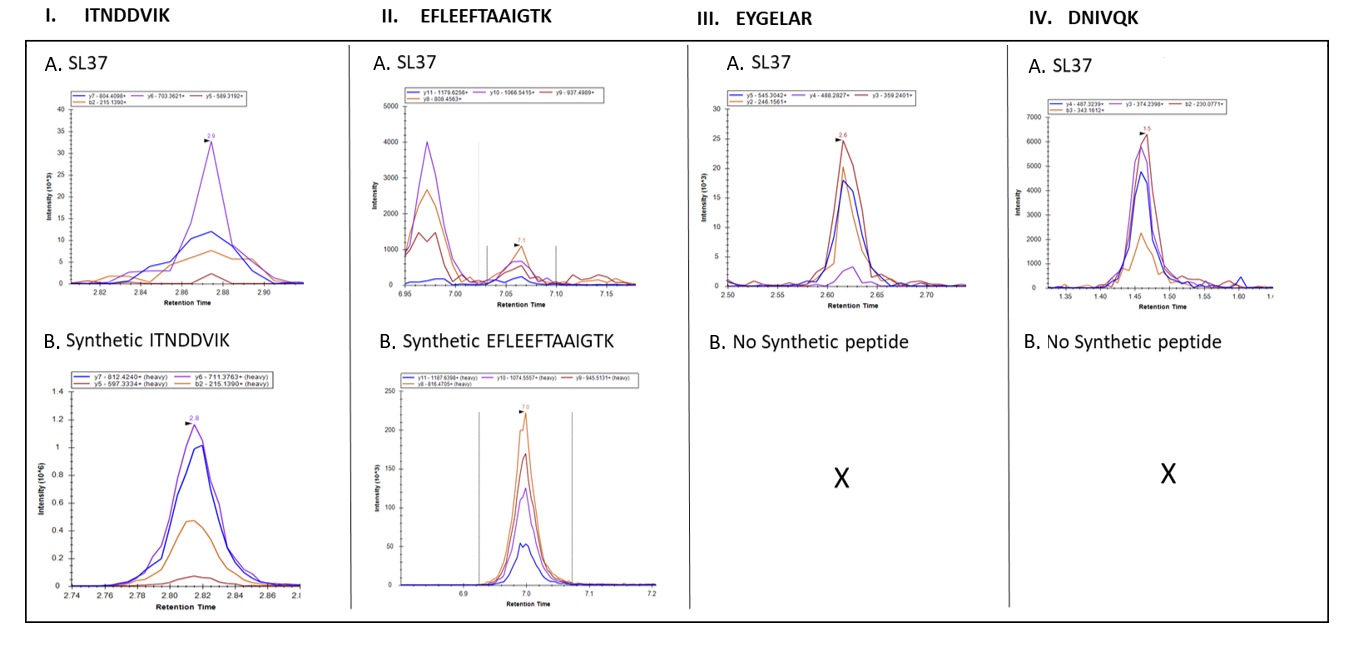
**

### **Supplementary Figure 9|** Extracted ion chromatograms (XIC) for four selected peptides (I) ITNDDVIK, (II) EFLEEFTAAIGTK, (III) EYGELAR and (IV) DNIVQK using MRM for AnfH.

The eluate after StrepTactin purification from leaves infiltrated with SL37 shows the presence of AnfH at a low level. (B) Two peptides (ITNDDVIK and EFLEEFTAAIGTK) were synthesised as heavy labelled peptides (JPT Peptide Technologies GmbH, Berlin, Germany) to distinguish interference peaks (e.g. EFLEEFTAAIGTK interference at RT= 6.95) and confirm that peptides detected displayed peak profiles with the expected product ion profile (Q3 m/z, n = 4), with the transition ions showing similar retention times (RT), 2.82 and 7.0, respectively. This supports the minimum threshold of protein identification with the identification of two unique AnfH peptides and is supported with the identification of ITNDDVIK using 6600TF LC-MS/MS in eluate SL37.

**AnfG**


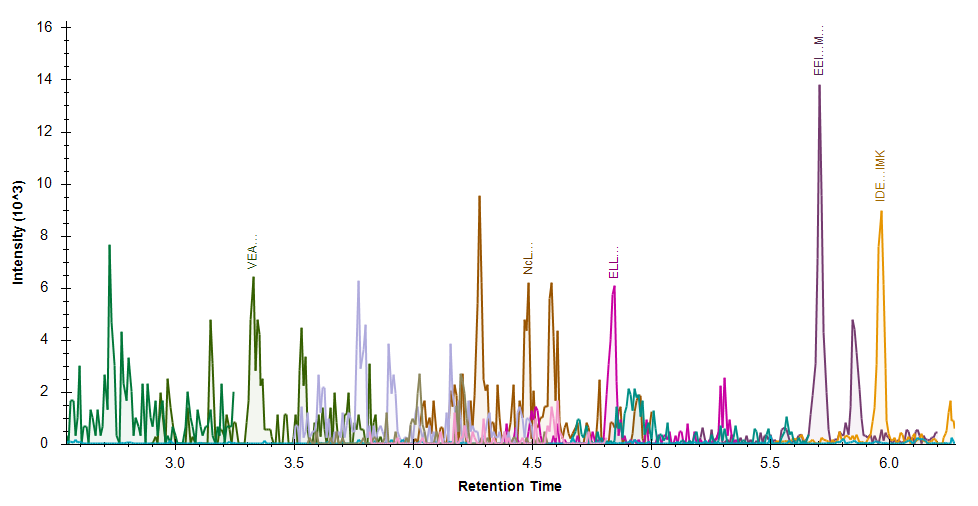


### **Supplementary Figure 10|** Extracted ion chromatogram (XIC) for AnfG tryptic peptides from the eluate of the StrepTactin purification from leaves infiltrated with SL37.

Seven MRM peptides were assessed for AnfG. Five out of seven peptides displayed peak profiles with low but detectable response and the product ions (Q3 m/z, n = 4) profiled with the same retention times.

**AnfG**


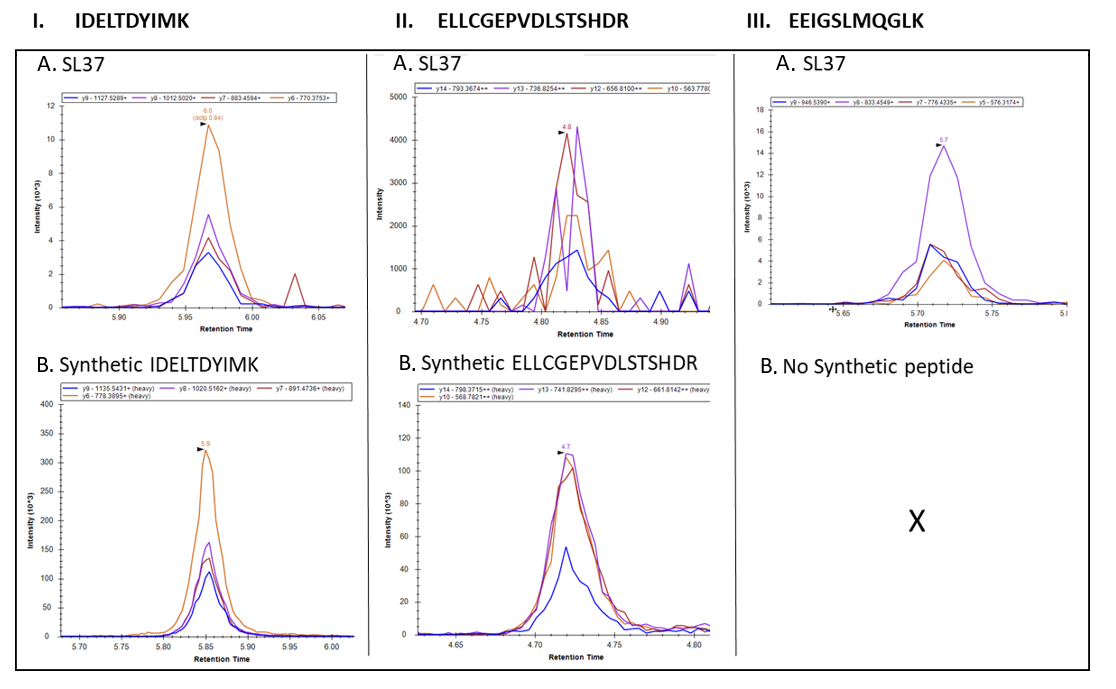


### **Supplementary Figure 11|** Extracted ion chromatograms (XIC) for three selected peptides; (I) IDELTDYIMK, (II) ELLCGEPVDLSTSHDR and (III) EEIGSLMQGLK using MRM for AnfG.

The eluate after streptactin purification from leaves infiltrated with SL37 shows very low level of AnfG. Two peptides, IDELTDYIMK and ELLCGEPVDLSTSHDR (B) were synthesised as heavy labelled peptides (JPT Peptide Technologies GmbH, Berlin, Germany) to distinguish interference peaks and confirm that both the peptide peak profiles display the expected product ion profile (Q3 m/z, n = 4), and the transition ions show similar retention times (RT), 5.9 and 4.7, respectively. There is evidence of matrix effects on both of these peptides with slight RT shifts between the dilute eluate (IDELTDYIMK, RT=6.0 and ELLCGEPVDLSTSHDR, RT=4.8) compared to the synthetic peptides spiked into total protein extract from *N. benthamiana* prior to the trypsin digest (B). The evidence for the presence of AnfG is not conclusive with the confirmation of the unique IDELTDYIMK AnfG peptide, but very low detection for ELLCGEPVDLSTSHDR (DNQ: detected but not quantifiable).

### **Supplementary Table 1|** Multiple reaction monitoring transitions of AnfG, H, D and K peptides for targeted liquid chromatography – mass spectrometry (LC-MS).

| **Protein** | **Peptide^a^** | **RT (min)^b^** | **Q1** | **z ^b^** | **Q3** | **Fragment** | **CE ^c^** |
| --- | --- | --- | --- | --- | --- | --- | --- |
|  |  |  | ***m/z* ^b^** |  | ***m/z* ^b^** |  |  |
|  |  |  |  |  | 229.12 | b2+ | 17.58 |
| AnfG | VEAPVHPMDAR | 3.39 | 407.87 | 3+ | 561.77 | y10++ | 17.58 |
|  |  |  |  |  | 589.28 | y5+ | 17.58 |
|  |  |  |  |  | 497.25 | y9++ | 17.58 |
|  |  |  |  |  | 229.12 | b2+ | 17.83 |
| AnfG | VEAPVHPM[Oxi]DAR | 2.66 | 413.20 | 3+ | 569.77 | y10++ | 17.83 |
|  |  |  |  |  | 469.73 | y5+ | 17.83 |
|  |  |  |  |  | 505.25 | y9++ | 17.83 |
|  |  |  |  |  | 770.38 | y6+ | 29.42 |
| AnfG | IDELTDYIMK | 6.02 | 620.81 | 2+ | 883.46 | y7+ | 29.42 |
|  |  |  |  |  | 1012.50 | y8+ | 29.42 |
|  |  |  |  |  | 1127.53 | y9+ | 29.42 |
|  |  |  |  |  | 786.37 | y6+ | 29.81 |
| AnfG | IDELTDYIM[Oxi]K | 5.16 | 628.81 | 2+ | 899.45 | y7+ | 29.81 |
|  |  |  |  |  | 1028.50 | y8+ | 29.81 |
|  |  |  |  |  | 1143.52 | y9+ | 29.81 |
|  |  |  |  |  | 399.21 | y3+ | 17.99 |
| AnfG | NC[CAM]LWQFHSR | 4.50 | 416.53 | 3+ | 674.34 | y5+ | 17.99 |
|  |  |  |  |  | 430.71 | y6++ | 17.99 |
|  |  |  |  |  | 487.25 | y7++ | 17.99 |
|  |  |  |  |  | 147.11 | y1+ | 19.00 |
| AnfG | QNAEILK | 2.74 | 408.23 | 2+ | 260.20 | y2+ | 19.00 |
|  |  |  |  |  | 373.28 | y3+ | 19.00 |
|  |  |  |  |  | 573.36 | y5+ | 19.00 |
|  |  |  |  |  | 147.11 | y1+ | 18.59 |
| AnfG | Q[PGQ]NAEILK | 4.00 | 399.72 | 2+ | 260.20 | y2+ | 18.59 |
|  |  |  |  |  | 373.28 | y3+ | 18.59 |
|  |  |  |  |  | 573.36 | y5+ | 18.59 |
|  |  |  |  |  | 563.78 | y10++ | 27.28 |
| AnfG | ELLC[CAM]GEPVDLSTSHDR | 4.87 | 609.96 | 3+ | 656.81 | y12++ | 27.28 |
|  |  |  |  |  | 736.83 | y13++ | 27.28 |
|  |  |  |  |  | 793.37 | y14++ | 27.28 |
|  |  |  |  |  | 267.11 | b2+ | 33.51 |
| AnfG | EHYPWINSM[Oxi]SK | 4.10 | 704.32 | 2+ | 430.17 | b3+ | 33.51 |
|  |  |  |  |  | 978.47 | y8+ | 33.51 |
|  |  |  |  |  | 1141.53 | y9+ | 33.51 |
|  |  |  |  |  | 576.32 | y5+ | 28.54 |
| AnfG | EEIGSLMQGLK | 5.70 | 602.82 | 2+ | 776.43 | y7+ | 28.54 |
|  |  |  |  |  | 833.45 | y8+ | 28.54 |
|  |  |  |  |  | 946.54 | y9+ | 28.54 |
|  |  |  |  |  | 792.43 | y7+ | 28.93 |
| AnfG | EEIGSLM[Oxi]QGLK | 4.31 | 610.81 | 2+ | 849.45 | y8+ | 28.93 |
|  |  |  |  |  | 962.53 | y9+ | 28.93 |
|  |  |  |  |  | 481.77 | y9++ | 28.93 |
|  |  |  |  |  | 171.11 | b2+ | 14.96 |
| AnfH | VAIYGK | 2.66 | 325.70 | 2+ | 367.20 | y3+ | 14.96 |
|  |  |  |  |  | 480.28 | y4+ | 14.96 |
|  |  |  |  |  | 551.32 | y5+ | 14.96 |
|  |  |  |  |  | 826.41 | y15++ | 27.47 |
| AnfH | STTTQNTAAALAYFHDK | 5.01 | 613.97 | 3+ | 709.33 | y5+ | 27.47 |
|  |  |  |  |  | 780.37 | y6+ | 27.47 |
|  |  |  |  |  | 1035.53 | y9+ | 27.47 |
|  |  |  |  |  | 415.19 | b7++ | 15.19 |
| AnfH | VFIHGC[CAM]DPK | 2.78 | 358.18 | 3+ | 244.17 | y2+ | 15.19 |
|  |  |  |  |  | 413.70 | y7++ | 15.19 |
|  |  |  |  |  | 487.23 | y8++ | 15.19 |
|  |  |  |  |  | 215.14 | b2+ | 21.50 |
| AnfH | ITNDDVIK | 2.85 | 459.25 | 2+ | 589.32 | y5+ | 21.50 |
|  |  |  |  |  | 703.36 | y6+ | 21.50 |
|  |  |  |  |  | 804.41 | y7+ | 21.50 |
|  |  |  |  |  | 536.22 | y4+ | 23.27 |
| AnfH | LGGIIC[CAM]NSR | 3.59 | 495.26 | 2+ | 649.31 | y5+ | 23.27 |
|  |  |  |  |  | 819.41 | y7+ | 23.27 |
|  |  |  |  |  | 876.44 | y8+ | 23.27 |
|  |  |  |  |  | 1066.54 | y10+ | 34.69 |
| AnfH | EFLEEFTAAIGTK | 7.06 | 728.37 | 2+ | 1179.63 | y11+ | 34.69 |
|  |  |  |  |  | 808.46 | y8+ | 34.69 |
|  |  |  |  |  | 937.50 | y9+ | 34.69 |
|  |  |  |  |  | 230.08 | b2+ | 16.58 |
| AnfH | DNIVQK | 1.39 | 358.70 | 2+ | 343.16 | b3+ | 16.58 |
|  |  |  |  |  | 374.24 | y3+ | 16.58 |
|  |  |  |  |  | 487.32 | y4+ | 16.58 |
|  |  |  |  |  | 246.16 | y2+ | 19.54 |
| AnfH | EYGELAR | 2.59 | 419.21 | 2+ | 359.24 | y3+ | 19.54 |
|  |  |  |  |  | 488.28 | y4+ | 19.54 |
|  |  |  |  |  | 545.30 | y5+ | 19.54 |
|  |  |  |  |  | 461.17 | b4+ | 19.10 |
| AnfH | E[PGE]YGELAR | 2.59 | 410.20 | 2+ | 246.16 | y2+ | 19.10 |
|  |  |  |  |  | 359.24 | y3+ | 19.10 |
|  |  |  |  |  | 545.30 | y5+ | 19.10 |
|  |  |  |  |  | 237.13 | b2+ | 20.65 |
| AnfD | HVIGTPMK | 2.60 | 441.75 | 2+ | 375.21 | y3+ | 20.65 |
|  |  |  |  |  | 646.36 | y6+ | 20.65 |
|  |  |  |  |  | 745.43 | y7+ | 20.65 |
|  |  |  |  |  | 237.13 | b2+ | 21.04 |
| AnfD | HVIGTPM[Oxi]K | 1.70 | 449.74 | 2+ | 508.29 | b5+ | 21.04 |
|  |  |  |  |  | 662.35 | y6+ | 21.04 |
|  |  |  |  |  | 761.42 | y7+ | 21.04 |
|  |  |  |  |  | 265.12 | b2+ | 22.56 |
| AnfD | YTYATDVK | 2.81 | 480.74 | 2+ | 533.29 | y5+ | 22.56 |
|  |  |  |  |  | 696.36 | y6+ | 22.56 |
|  |  |  |  |  | 797.40 | y7+ | 22.56 |
|  |  |  |  |  | 251.15 | b2+ | 21.09 |
| AnfD | HIVFGAEK | 3.07 | 450.75 | 2+ | 551.28 | y5+ | 21.09 |
|  |  |  |  |  | 650.35 | y6+ | 21.09 |
|  |  |  |  |  | 763.43 | y7+ | 21.09 |
|  |  |  |  |  | 365.22 | y3+ | 22.61 |
| AnfD | QNIIEAFK | 5.16 | 481.77 | 2+ | 494.26 | y4+ | 22.61 |
|  |  |  |  |  | 607.34 | y5+ | 22.61 |
|  |  |  |  |  | 720.43 | y6+ | 22.61 |
|  |  |  |  |  | 228.13 | b2+ | 25.97 |
| AnfD | INIAWINQK | 5.75 | 550.32 | 2+ | 688.38 | y5+ | 25.97 |
|  |  |  |  |  | 759.41 | y6+ | 25.97 |
|  |  |  |  |  | 872.50 | y7+ | 25.97 |
|  |  |  |  |  | 436.23 | b4+ | 16.95 |
| AnfD | AHLNVLEC[CAM]AR | 3.81 | 394.87 | 3+ | 535.30 | b5+ | 16.95 |
|  |  |  |  |  | 406.19 | y3+ | 16.95 |
|  |  |  |  |  | 535.23 | y4+ | 16.95 |
|  |  |  |  |  | 711.87 | y13++ | 26.26 |
| AnfD | LDIDGFGFKPLADSLR | 7.48 | 588.65 | 3+ | 768.41 | y14++ | 26.26 |
|  |  |  |  |  | 825.93 | y15++ | 26.26 |
|  |  |  |  |  | 771.44 | y7+ | 26.26 |
|  |  |  |  |  | 589.29 | y5+ | 28.05 |
| AnfD | IGMFFGIEDR | 6.87 | 592.79 | 2+ | 736.36 | y6+ | 28.05 |
|  |  |  |  |  | 883.43 | y7+ | 28.05 |
|  |  |  |  |  | 1071.49 | y9+ | 28.05 |
|  |  |  |  |  | 589.29 | y5+ | 28.44 |
| AnfD | IGM[Oxi]FFGIEDR | 5.90 | 600.79 | 2+ | 736.36 | y6+ | 28.44 |
|  |  |  |  |  | 883.43 | y7+ | 28.44 |
|  |  |  |  |  | 1087.49 | y9+ | 28.44 |
|  |  |  |  |  | 185.13 | b2+ | 23.91 |
| AnfD | AIIDEEVAR | 3.89 | 508.27 | 2+ | 603.31 | y5+ | 23.91 |
|  |  |  |  |  | 718.34 | y6+ | 23.91 |
|  |  |  |  |  | 831.42 | y7+ | 23.91 |
|  |  |  |  |  | 496.26 | y3+ | 18.27 |
| AnfD | WKPELDWYK | 5.64 | 422.22 | 3+ | 611.28 | y4+ | 18.27 |
|  |  |  |  |  | 950.46 | y7+ | 18.27 |
|  |  |  |  |  | 539.78 | y8++ | 18.27 |
|  |  |  |  |  | 260.11 | b2+ | 23.61 |
| AnfD | VC[CAM]LWPGGSK | 4.86 | 502.26 | 2+ | 445.24 | y5+ | 23.61 |
|  |  |  |  |  | 631.32 | y6+ | 23.61 |
|  |  |  |  |  | 744.40 | y7+ | 23.61 |
|  |  |  |  |  | 199.14 | b2+ | 18.51 |
| AnfD | VVSVYTK | 2.74 | 398.23 | 2+ | 411.22 | y3+ | 18.51 |
|  |  |  |  |  | 597.32 | y5+ | 18.51 |
|  |  |  |  |  | 696.39 | y6+ | 18.51 |
|  |  |  |  |  | 205.10 | b2+ | 19.86 |
| AnfD | GFEGWVR | 4.75 | 425.71 | 2+ | 460.27 | y3+ | 19.86 |
|  |  |  |  |  | 517.29 | y4+ | 19.86 |
|  |  |  |  |  | 646.33 | y5+ | 19.86 |
|  |  |  |  |  | 451.23 | y3+ | 21.35 |
| AnfD | EYPAFER | 3.63 | 456.22 | 2+ | 522.27 | y4+ | 21.35 |
|  |  |  |  |  | 619.32 | y5+ | 21.35 |
|  |  |  |  |  | 310.16 | y5++ | 21.35 |
|  |  |  |  |  | 227.18 | b2+ | 14.60 |
| AnfK | LIFSQHYK | 3.79 | 345.86 | 3+ | 662.33 | y5+ | 14.60 |
|  |  |  |  |  | 405.20 | y6++ | 14.60 |
|  |  |  |  |  | 461.74 | y7++ | 14.60 |
|  |  |  |  |  | 702.41 | y6+ | 29.15 |
| AnfK | VEEAVDVLLSR | 5.94 | 615.34 | 2+ | 801.48 | y7+ | 29.15 |
|  |  |  |  |  | 872.52 | y8+ | 29.15 |
|  |  |  |  |  | 1001.56 | y9+ | 29.15 |
|  |  |  |  |  | 376.15 | b3+ | 14.25 |
| AnfK | YPDVK | 2.07 | 311.17 | 2+ | 246.18 | y2+ | 14.25 |
|  |  |  |  |  | 361.21 | y3+ | 14.25 |
|  |  |  |  |  | 458.26 | y4+ | 14.25 |
|  |  |  |  |  | 228.13 | b2+ | 18.29 |
| AnfK | LNEGLLK | 3.85 | 393.74 | 2+ | 430.30 | y4+ | 18.29 |
|  |  |  |  |  | 559.34 | y5+ | 18.29 |
|  |  |  |  |  | 673.39 | y6+ | 18.29 |
|  |  |  |  |  | 228.13 | b2+ | 36.41 |
| AnfK | INLLTGWVNPGDVK | 7.47 | 763.42 | 2+ | 341.22 | b3+ | 36.41 |
|  |  |  |  |  | 1072.54 | y10+ | 36.41 |
|  |  |  |  |  | 971.49 | y9+ | 36.41 |
|  |  |  |  |  | 402.25 | y3+ | 18.44 |
| AnfK | ATFALNR | 3.67 | 396.72 | 2+ | 473.28 | y4+ | 18.44 |
|  |  |  |  |  | 620.35 | y5+ | 18.44 |
|  |  |  |  |  | 310.68 | y5++ | 18.44 |
|  |  |  |  |  | 143.08 | b2+ | 19.17 |
| AnfK | AAEYLQK | 2.21 | 411.72 | 2+ | 551.32 | y4+ | 19.17 |
|  |  |  |  |  | 680.36 | y5+ | 19.17 |
|  |  |  |  |  | 751.40 | y6+ | 19.17 |
|  |  |  |  |  | 1204.74 | y12+ | 38.08 |
| AnfK | FEIPAIIGPTPIGIR | 8.55 | 797.47 | 2+ | 753.46 | y7+ | 38.08 |
|  |  |  |  |  | 810.48 | y8+ | 38.08 |
|  |  |  |  |  | 923.57 | y9+ | 38.08 |
|  |  |  |  |  | 615.38 | y5+ | 28.56 |
| AnfK | NTDIFLQNLK | 5.88 | 603.33 | 2+ | 762.45 | y6+ | 28.56 |
|  |  |  |  |  | 875.53 | y7+ | 28.56 |
|  |  |  |  |  | 990.56 | y8+ | 28.56 |
|  |  |  |  |  | 263.14 | b2+ | 17.75 |
| AnfK | YVDDPR | 1.43 | 382.68 | 2+ | 493.19 | b4+ | 17.75 |
|  |  |  |  |  | 272.17 | y2+ | 17.75 |
|  |  |  |  |  | 502.23 | y4+ | 17.75 |
|  |  |  |  |  | 647.87 | y12++ | 22.64 |
| AnfK | NEGLELDLILGHSK | 6.72 | 513.28 | 3+ | 428.23 | y4+ | 22.64 |
|  |  |  |  |  | 541.31 | y5+ | 22.64 |
|  |  |  |  |  | 654.39 | y6+ | 22.64 |
|  |  |  |  |  | 1221.63 | y10+ | 35.33 |
| AnfK | FISIDYNIPMLR | 7.81 | 741.39 | 2+ | 516.30 | y4+ | 35.33 |
|  |  |  |  |  | 906.49 | y7+ | 35.33 |
|  |  |  |  |  | 1021.51 | y8+ | 35.33 |
|  |  |  |  |  | 1237.62 | y10+ | 35.72 |
| AnfK | FISIDYNIPM[Oxi]LR | 7.12 | 749.39 | 2+ | 532.29 | y4+ | 35.72 |
|  |  |  |  |  | 922.48 | y7+ | 35.72 |
|  |  |  |  |  | 1037.51 | y8+ | 35.72 |
|  |  |  |  |  | 651.31 | y5+ | 22.41 |
| AnfK | VGFPTYDR | 4.18 | 477.74 | 2+ | 326.16 | y5++ | 22.41 |
|  |  |  |  |  | 399.69 | y6++ | 22.41 |
|  |  |  |  |  | 855.40 | y7+ | 22.41 |

1. The peptide sequence is represented by single amino acid code. C[CAM] refers to carbamidomethylation of cysteine; M[Oxi] refers to oxidation of methionine; E[PGE] refers to pyroglutamic acid formed from glutamic acid.
2. RT, retention time (min); Q1 *m/z*, precursor ion mass-to-charge ratio; z, charge state; Q3 *m/z*, fragment ion *m/z*; CE, collision energy in V.
3. Collision energy settings were calculated for 2+ ions (CE = slope (0.049) x (precursor *m/z*) + intercept (-1.0)) and 3+ ions (CE = slope (0.048) x (precursor *m/z*) + intercept (-2.0)) for a 6500 QTRAP mass spectrometer (SCIEX, Redwood City, USA).

### **Supplementary Data Set 1|** Schematic outline of the peptides detected by LC-MS/MS for AnfDKGH isolated from a range of plant-based expression experiments

Using a variety of protein sources expressed from multigene and single gene constructs (Table 1, Table 2), the Anf proteins were characterized allowing selection of peptides for MRM development. Green, peptides identified with >95% confidence; yellow, peptides identified with 50-95% confidence; red, peptides identified with <50% confidence; grey, not detected. Peptides used for MRM are underlined. Solid and dashed underline are used to distinguish adjacent tryptic peptides (5-20 amino acids in length) in the MRM method.

**AnfD**

MPHHEFECSK**VIPER**KKHAVIK**GKGETLADALPQGYLNTIPGSISER**GCAYCGAK**HVIGTPMKDVIHISHGPVGCTYDTWQTKRYISDNDNFQLKYTYATDVK**EK**HIVFGAEK**LLK**QNIIEAFKAFPQIK**RMTIYQTCATALIGDDINAIAEEVMEEMPEVDIF**VCNSPGFAGPSQSGGHHKINIAWINQK**VGTVEPEITGDHVINYVGEYNIQ**GDQEVMVDYFK**RMGIQVL**STFTGNGSYDGLR**AMHR**AHLNVLECARSAEYICNELR**VR**YGIPRLDIDGFGFKPLADSLRKIGMFFGIEDR**AK**AIIDEEVARWKPELDWYKER**LMGK**KVCLWPGGSKLWHWAHVIEEEMGLKVVSVYTKFGHQGDMEK**GIARCGEGTLAIDDPNELEGLEALEM**LKPDIILTGKRPGEVAK**KVR**VPYLNAHAYHNGPYKGFEGWVR**FAR**DIYNAIYSPIHQLSGIDITKDNAPEWGNGFR**TR**QMLSDGNLSDAVR**NSETLR**QYTGGYDSVSKL**R**EREYPAFERK**VG

**AnfK**

MTCEVKEKGR**VGTINPIFTCQPAGAQFVSIGIKDCIGIVHGGQGCVMFVRLIFSQHYKESFELASSSLHEDGAVFGACGRVEEAVDVLLSRYPDVKVVPIITTCSTEIIGDDVDGVIKKLNEGLLKEKFPDREVHLIAMHTPSFVGSMISGYDVAVR**DVVR**HFAKREAPNDKINLLTGWVNPGDVKELKHLLGEMDIEANVLFEIESFDSPILPDGSAVSHGNTTIEDLIDTGNARATFALNRYEGTKAAEYLQKKFEIPAIIGPTPIGIRNTDIFLQNLKKATGKPIPQSLAHERGVAIDALADLTHMFLAEKRVAIYGAPDLVIGLA**EF**CLDLEMKPVLLLLGDDNSKYVDDPR**IK**ALQENVDYGMEIVTNADFWELENRIKNEGLELDLILGHSKG**R**FISIDYNIPMLRVGFPTYDR**AGLFRYPTVGYGGAIW**LAEQMANTLFADMEHKKNKEWVLNVW**

**AnfH**

**MTRKVAIYGKG**GIGK**STTTQNTAAALAYFHDKKVFIHGCDPK**ADSTR**LILGGK**PQETLMDMLRDKGAEK**ITNDDVIKKGFLDIQCVESGGPEPGVGCAGR**GVITAIDLMEENGAYTDDLDFVFFDVLGDVVCGGFAMPIRDGKAQEVYIVASGEMMAIYAANNICKGLVKYAKQSGVR**LGGIICNSRK**VDGER**EFLEEFTAAIGTKMIHFVPRDNIVQK**AEFNKK**TVTEFAPEENQAKEYGELARKIIENDEFVIPKPL**TMDQLEDMVVKYGIAD

**AnfG**

MSTASAAAVVK**QKVEAPVHPMDARIDELTDYIMKNCLWQFHSR**SWDRER**QNAEILKK**TK**ELLCGEPVDLSTSHDR**CYWVDAVCLADDYR**EHYPWINSMSKEEIGSLMQGLK**DR**MDYLTITGSLNEELSDK**HY

### **Supplementary Data Set 2|** Schematic outline of the peptides detected by LC-MS/MS for MTP-AnfDKH from enriched plant mitochondria.

Duplicate gel slices of protein bands corresponding to AnfD, K, H and G were excised from Coomassie stained gels and subjected to in-gel digestion (IGD) with trypsin. Peptide identifications and the sequence coverage is shown for AnfH (IGD_9-1 and -2), AnfK (IGD_10-1 and -2) and AnfD (IGD_11-1 and ‑2). The duplicate gel digests (-1 or -2) show similar peptide identifications. No peptides were identified for AnfG in either digest. Green, peptides identified with >95% confidence; yellow, peptides identified with 50-95% confidence; red, peptides identified with <50% confidence; grey, not detected.

IGD_9-1. pFAγ51-HA-AnfH (3 peptides were identified at 95% confidence, 10% sequence coverage).

MAMAVFRREGRRLLPSIAARPIAAIRSPLSSDQEEGLLGVRSISTQVVRNRGGYPYDVPDYAGGMTRKVAIYGKGGIGKSTTTQNTAAALAYFHDKKVFIHGCDPKADSTRLILGGKPQETLMDMLRDKGAEK**ITNDDVIK**KGFLDIQCVESGGPEPGVGCAGRGVITAIDLMEENGAYTDDLDFVFFDVLGDVVCGGFAMPIRDGKAQEVYIVASGEMMAIYAANNICKGLVKYAKQSGVRLGGIICNSRKVDGER**EFLEEFTAAIGTK**MIHFVPRDNIVQKAEFNKK**TVTEFAPEENQAK**EYGELARKIIENDEFVIPKPLTMDQLEDMVVKYGIAD

IGD_9-2. pFAγ51-HA-AnfH (3 peptides were identified at 95% confidence, 9.7% sequence coverage).

MAMAVFRREGRRLLPSIAARPIAAIRSPLSSDQEEGLLGVRS**ISTQVVR**NRGGYPYDVPDYAGGMTRKVAIYGKGGIGKSTTTQNTAAALAYFHDKKVFIHGCDPKADSTRLILGGKPQETLMDMLRDKGAEKITNDDVIKKGFLDIQCVESGGPEPGVGCAGRGVITAIDLMEENGAYTDDLDFVFFDVLGDVVCGGFAMPIRDGKAQEVYIVASGEMMAIYAANNICKGLVKYAKQSGVRLGGIICNSRKVDGER**EFLEEFTAAIGTK**MIHFVPRDNIVQKAEFNKK**TVTEFAPEENQAK**EYGELARKIIENDEFVIPKPLTMDQLEDMVVKYGIAD

IGD_10-1. pFAγ51-HA-AnfK (5 peptides were identified at 95% confidence, 10.8% sequence coverage).

MAMAVFRREGRRLLPSIAARPIAAIRSPLSSDQEEGLLGVRSISTQVVRNRGGYPYDVPDYAGGMTCEVKEKGRVGTINPIFTCQPAGAQFVSIGIKDCIGIVHGGQGCVMFVR**LIFSQHYK**ESFELASSSLHEDGAVFGACGRVEEAVDVLLSRYPDVKVVPIITTCSTEIIGDDVDGVIKKLNEGLLKEKFPDREVHLIAMHTPSFVGSMISGYDVAVRDVVRHFAKREAPNDK**INLLTGWVNPGDVK**ELKHLLGEMDIEANVLFEIESFDSPILPDGSAVSHGNTTIEDLIDTGNAR**ATFALNR**YEGTK**AAEYLQKKFEIPAIIGPTPIGIR**NTDIFLQNLKKATGKPIPQSLAHERGVAIDALADLTHMFLAEKRVAIYGAPDLVIGLAEFCLDLEMKPVLLLLGDDNSK**YVDDPR**IKALQENVDYGMEIVTNADFWELENRIKNEGLELDLILGHSKGR**FISIDYNIPMLRVGFPTYDR**AGLFRYPTVGYGGAIWLAEQMANTLFADMEHKKNKEWVLNVW

IGD_10-2. pFAγ51-HA-AnfK (4 peptides were identified at 95% confidence, 10.1% sequence coverage).

MAMAVFRREGRRLLPSIAARPIAAIRSPLSSDQEEGLLGVRSISTQVVRNRGGYPYDVPDYAGGMTCEVKEKGRVGTINPIFTCQPAGAQFVSIGIKDCIGIVHGGQGCVMFVRLIFSQHYKESFELASSSLHEDGAVFGACGR**VEEAVDVLLSR**YPDVKVVPIITTCSTEIIGDDVDGVIKKLNEGLLKEKFPDREVHLIAMHTPSFVGSMISGYDVAVRDVVRHFAKREAPNDK**INLLTGWVNPGDVK**ELKHLLGEMDIEANVLFEIESFDSPILPDGSAVSHGNTTIEDLIDTGNAR**ATFALNR**YEGTKAAEYLQK**KFEIPAIIGPTPIGIR**NTDIFLQNLKKATGKPIPQSLAHERGVAIDALADLTHMFLAEKRVAIYGAPDLVIGLAEFCLDLEMKPVLLLLGDDNSKYVDDPRIKALQENVDYGMEIVTNADFWELENRIKNEGLELDLILGHSKGR**FISIDYNIPMLRVGFPTYDR**AGLFRYPTVGYGGAIWLAEQMANTLFADMEHKKNKEWVLNVW

IGD_11-1. pFAγ51-HA-AnfD (9 peptides were identified at 95% confidence, 17.0% sequence coverage).

MAMAVFRREGRRLLPSIAARPIAAIRSPLSSDQEEGLLGVRS**ISTQVVR**NRGGYPYDVPDYAGGMPHHEFECSKVIPERKKHAVIKGKGETLADALPQGYLNTIPGSISERGCAYCGAK**HVIGTPMK**DVIHISHGPVGCTYDTWQTKR**YISDNDNFQLKYTYATDVK**EKHIVFGAEKLLK**QNIIEAFK**AFPQIKRMTIYQTCATALIGDDINAIAEEVMEEMPEVDIFVCNSPGFAGPSQSGGHHK**INIAWINQK**VGTVEPEITGDHVINYVGEYNIQGDQEVMVDYFKRMGIQVLSTFTGNGSYDGLRAMHRAHLNVLECAR**SAEYICNELR**VRYGIPRLDIDGFGFKPLADSLRKIGMFFGIEDRAKAIIDEEVARWKPELDWYKERLMGKKVCLWPGGSKLWHWAH**VIEEEMGLKVVSVYTK**FGHQGDMEKGIARCGEGTLAIDDPNELEGLEALEMLKPDIILTGKRPGEVAKKVRVPYLNAHAYHNGPYK**GFEGWVR**FAR**DIYNAIYSPIHQLSGIDITKDNAPEWGNGFR**TR**QMLSDGNLSDAVR**NSETLR**QYTGGYDSVSK**LREREYPAFERKVG

IGD_11-2. pFAγ51-HA-AnfD (10 peptides were identified at 95% confidence, 18.9% sequence coverage).

MAMAVFRREGRRLLPSIAARPIAAIRSPLSSDQEEGLLGVRS**ISTQVVR**NRGGYPYDVPDYAGGMPHHEFECSKVIPERKKHAVIK**GKGETLADALPQGYLNTIPGSISER**GCAYCGAKHVIGTPMKDVIHISHGPVGCTYDTWQTKR**YISDNDNFQLKYTYATDVK**EK**HIVFGAEK**LLK**QNIIEAFK**AFPQIKRMTIYQTCATALIGDDINAIAEEVMEEMPEVDIFVCNSPGFAGPSQSGGHHKINIAWINQKVGTVEPEITGDHVINYVGEYNIQGDQEVMVDYFKRMGIQVLSTFTGNGSYDGLRAMHRAHLNVLECAR**SAEYICNELR**VRYGIPR**LDIDGFGFKPLADSLR**KIGMFFGIEDRAK**AIIDEEVAR**WKPELDWYKERLMGKKVCLWPGGSKLWHWAHVIEEEMGLK**VVSVYTK**FGHQGDMEKGIARCGEGTLAIDDPNELEGLEALEMLKPDIILTGKRPGEVAKKVRVPYLNAHAYHNGPYK**GFEGWVR**FAR**DIYNAIYSPIHQLSGIDITKDNAPEWGNGFR**TR**QMLSDGNLSDAVR**NSETLR**QYTGGYDSVSK**LREREYPAFERKVG

### **Supplementary Data Set 3|**Codon-optimized DNA sequences and translated protein sequences of *anfD*, *anfK*, *anfG* and *anfH* genes used in this study.

Amino acids underlined represent the MTP sequence, amino acids in **bold** represent epitope tags, HA and Twin-Strep.

**SN81, pFAγ51-AnfD-HA**:

atgatggcaatggctgttttccgtcgcgaagggaggcgtctcctcccttcaatcgccgct
 M  M  A  M  A  V  F  R  R  E  G  R  R  L  L  P  S  I  A  A 
cgcccaatcgctgctatccgatctcctctctcttctgaccaggaggaaggacttcttgga
 R  P  I  A  A  I  R  S  P  L  S  S  D  Q  E  E  G  L  L  G 
gttcgatctatctcaactcaagtggtgcgtaaccgcggaggtatgcctcatcatgagttc
 V  R  S  I  S  T  Q  V  V  R  N  R  G  G  M  P  H  H  E  F 
gagtgttccaaagtaatcccggagagaaagaagcatgccgttataaaaggaaagggcgag
 E  C  S  K  V  I  P  E  R  K  K  H  A  V  I  K  G  K  G  E 
acactagcggacgcgcttcctcaaggatatctaaacacaatacctgggtcaatttccgaa
 T  L  A  D  A  L  P  Q  G  Y  L  N  T  I  P  G  S  I  S  E 
agaggttgtgcttattgcggtgcaaagcacgttattggaaccccaatgaaagatgttata
 R  G  C  A  Y  C  G  A  K  H  V  I  G  T  P  M  K  D  V  I 
cacatttcccacggaccagttggctgtacttacgatacatggcagacgaaacgttacata
 H  I  S  H  G  P  V  G  C  T  Y  D  T  W  Q  T  K  R  Y  I 
tccgacaatgacaatttccagctaaagtatacttatgcgacggacgtaaaagaaaaacac
 S  D  N  D  N  F  Q  L  K  Y  T  Y  A  T  D  V  K  E  K  H 
atagtctttggagctgaaaaattactgaaacaaaacatcattgaggcgtttaaggcattc
 I  V  F  G  A  E  K  L  L  K  Q  N  I  I  E  A  F  K  A  F 
ccgcaaatcaagcgaatgactatctaccagacttgtgcgactgctttgatcggagatgat
 P  Q  I  K  R  M  T  I  Y  Q  T  C  A  T  A  L  I  G  D  D 
attaatgccatcgcggaagaggtaatggaggagatgcctgaagttgacatatttgtttgc
 I  N  A  I  A  E  E  V  M  E  E  M  P  E  V  D  I  F  V  C 
aactctcccggattcgctggaccatctcagagtggcgggcatcacaagatcaatattgcc
 N  S  P  G  F  A  G  P  S  Q  S  G  G  H  H  K  I  N  I  A 
tggattaatcagaaggtcggcacagtagagcctgaaattaccggtgaccatgtcataaac
 W  I  N  Q  K  V  G  T  V  E  P  E  I  T  G  D  H  V  I  N 
tatgtgggggaatataacatacaaggcgaccaggaggttatggttgactactttaagaga
 Y  V  G  E  Y  N  I  Q  G  D  Q  E  V  M  V  D  Y  F  K  R 
atggggatccaggtcttgtccactttcaccggcaacggaagttatgatgggttgagagct
 M  G  I  Q  V  L  S  T  F  T  G  N  G  S  Y  D  G  L  R  A 
atgcaccgtgcgcacttgaatgtattagagtgtgctcgtagcgcagagtacatttgcaat
 M  H  R  A  H  L  N  V  L  E  C  A  R  S  A  E  Y  I  C  N 
gaactaagagttagatacggcatccctcgacttgatatagacggttttggattcaaacca
 E  L  R  V  R  Y  G  I  P  R  L  D  I  D  G  F  G  F  K  P 
cttgcggactcactccgtaagattggaatgttctttgggatagaggaccgtgcaaaagcc
 L  A  D  S  L  R  K  I  G  M  F  F  G  I  E  D  R  A  K  A 
atcatcgacgaggaagtagctcgttggaaaccggaattggattggtataaggagcgactc
 I  I  D  E  E  V  A  R  W  K  P  E  L  D  W  Y  K  E  R  L 
atgggcaagaaggtgtgcctatggcctggtggctccaaattgtggcattgggcgcacgtc
 M  G  K  K  V  C  L  W  P  G  G  S  K  L  W  H  W  A  H  V 
atcgaggaagaaatgggattgaaggtcgtgtccgtctacaccaagttcggacatcaaggc
 I  E  E  E  M  G  L  K  V  V  S  V  Y  T  K  F  G  H  Q  G 
gacatggagaagggaatagcccgatgtggcgaaggtactcttgcgatcgatgatccaaat
 D  M  E  K  G  I  A  R  C  G  E  G  T  L  A  I  D  D  P  N 
gaactcgaaggtttagaagctcttgagatgcttaaaccagatataattttgacgggaaag
 E  L  E  G  L  E  A  L  E  M  L  K  P  D  I  I  L  T  G  K 
cgtccaggtgaggtggcaaaaaaagtccgtgttccttacctcaatgcccacgcttaccac
 R  P  G  E  V  A  K  K  V  R  V  P  Y  L  N  A  H  A  Y  H 
aacgggccttacaaaggttttgaaggttgggtgcgttttgctagggatatttataatgct
 N  G  P  Y  K  G  F  E  G  W  V  R  F  A  R  D  I  Y  N  A 
atctacagtccaatccatcagctgtccggaatcgatataactaaggataatgcccctgaa
 I  Y  S  P  I  H  Q  L  S  G  I  D  I  T  K  D  N  A  P  E 
tgggggaatggctttcgtacgcgacagatgctcagtgacgggaatttgagcgacgctgtc
 W  G  N  G  F  R  T  R  Q  M  L  S  D  G  N  L  S  D  A  V 
aggaattcagaaacactgcgtcagtacacaggcggttatgactctgtatcaaaactgcga
 R  N  S  E  T  L  R  Q  Y  T  G  G  Y  D  S  V  S  K  L  R 
gaaagagagtatcccgcgtttgaacgtaaggttggtggtggctacccttacgacgttcct
 E  R  E  Y  P  A  F  E  R  K  V  G  G  G  **Y  P  Y  D  V  P** 
gattacgct
 **D  Y  A**

**SN82, HA-AnfD:**

atgtacccttacgacgttcctgattacgctggaggtatgcctcatcatgagttcgagtgt
 M  **Y  P  Y  D  V  P  D  Y**  A  G  G  M  P  H  H  E  F  E  C 
tccaaagtaatcccggagagaaagaagcatgccgttataaaaggaaagggcgagacacta
 S  K  V  I  P  E  R  K  K  H  A  V  I  K  G  K  G  E  T  L 
gcggacgcgcttcctcaaggatatctaaacacaatacctgggtcaatttccgaaagaggt
 A  D  A  L  P  Q  G  Y  L  N  T  I  P  G  S  I  S  E  R  G 
tgtgcttattgcggtgcaaagcacgttattggaaccccaatgaaagatgttatacacatt
 C  A  Y  C  G  A  K  H  V  I  G  T  P  M  K  D  V  I  H  I 
tcccacggaccagttggctgtacttacgatacatggcagacgaaacgttacatatccgac
 S  H  G  P  V  G  C  T  Y  D  T  W  Q  T  K  R  Y  I  S  D 
aatgacaatttccagctaaagtatacttatgcgacggacgtaaaagaaaaacacatagtc
 N  D  N  F  Q  L  K  Y  T  Y  A  T  D  V  K  E  K  H  I  V 
tttggagctgaaaaattactgaaacaaaacatcattgaggcgtttaaggcattcccgcaa
 F  G  A  E  K  L  L  K  Q  N  I  I  E  A  F  K  A  F  P  Q 
atcaagcgaatgactatctaccagacttgtgcgactgctttgatcggagatgatattaat
 I  K  R  M  T  I  Y  Q  T  C  A  T  A  L  I  G  D  D  I  N 
gccatcgcggaagaggtaatggaggagatgcctgaagttgacatatttgtttgcaactct
 A  I  A  E  E  V  M  E  E  M  P  E  V  D  I  F  V  C  N  S 
cccggattcgctggaccatctcagagtggcgggcatcacaagatcaatattgcctggatt
 P  G  F  A  G  P  S  Q  S  G  G  H  H  K  I  N  I  A  W  I 
aatcagaaggtcggcacagtagagcctgaaattaccggtgaccatgtcataaactatgtg
 N  Q  K  V  G  T  V  E  P  E  I  T  G  D  H  V  I  N  Y  V 
ggggaatataacatacaaggcgaccaggaggttatggttgactactttaagagaatgggg
 G  E  Y  N  I  Q  G  D  Q  E  V  M  V  D  Y  F  K  R  M  G 
atccaggtcttgtccactttcaccggcaacggaagttatgatgggttgagagctatgcac
 I  Q  V  L  S  T  F  T  G  N  G  S  Y  D  G  L  R  A  M  H 
cgtgcgcacttgaatgtattagagtgtgctcgtagcgcagagtacatttgcaatgaacta
 R  A  H  L  N  V  L  E  C  A  R  S  A  E  Y  I  C  N  E  L 
agagttagatacggcatccctcgacttgatatagacggttttggattcaaaccacttgcg
 R  V  R  Y  G  I  P  R  L  D  I  D  G  F  G  F  K  P  L  A 
gactcactccgtaagattggaatgttctttgggatagaggaccgtgcaaaagccatcatc
 D  S  L  R  K  I  G  M  F  F  G  I  E  D  R  A  K  A  I  I 
gacgaggaagtagctcgttggaaaccggaattggattggtataaggagcgactcatgggc
 D  E  E  V  A  R  W  K  P  E  L  D  W  Y  K  E  R  L  M  G 
aagaaggtgtgcctatggcctggtggctccaaattgtggcattgggcgcacgtcatcgag
 K  K  V  C  L  W  P  G  G  S  K  L  W  H  W  A  H  V  I  E 
gaagaaatgggattgaaggtcgtgtccgtctacaccaagttcggacatcaaggcgacatg
 E  E  M  G  L  K  V  V  S  V  Y  T  K  F  G  H  Q  G  D  M 
gagaagggaatagcccgatgtggcgaaggtactcttgcgatcgatgatccaaatgaactc
 E  K  G  I  A  R  C  G  E  G  T  L  A  I  D  D  P  N  E  L 
gaaggtttagaagctcttgagatgcttaaaccagatataattttgacgggaaagcgtcca
 E  G  L  E  A  L  E  M  L  K  P  D  I  I  L  T  G  K  R  P 
ggtgaggtggcaaaaaaagtccgtgttccttacctcaatgcccacgcttaccacaacggg
 G  E  V  A  K  K  V  R  V  P  Y  L  N  A  H  A  Y  H  N  G 
ccttacaaaggttttgaaggttgggtgcgttttgctagggatatttataatgctatctac
 P  Y  K  G  F  E  G  W  V  R  F  A  R  D  I  Y  N  A  I  Y 
agtccaatccatcagctgtccggaatcgatataactaaggataatgcccctgaatggggg
 S  P  I  H  Q  L  S  G  I  D  I  T  K  D  N  A  P  E  W  G 
aatggctttcgtacgcgacagatgctcagtgacgggaatttgagcgacgctgtcaggaat
 N  G  F  R  T  R  Q  M  L  S  D  G  N  L  S  D  A  V  R  N 
tcagaaacactgcgtcagtacacaggcggttatgactctgtatcaaaactgcgagaaaga
 S  E  T  L  R  Q  Y  T  G  G  Y  D  S  V  S  K  L  R  E  R 
gagtatcccgcgtttgaacgtaaggttggt
 E  Y  P  A  F  E  R  K  V  G

**SN129, pFAγ51-HA-AnfK:**

atggcaatggctgttttccgtcgcgaagggaggcgtctcctcccttcaatcgccgctcgc
 M  A  M  A  V  F  R  R  E  G  R  R  L  L  P  S  I  A  A  R 
ccaatcgctgctatccgatctcctctctcttctgaccaggaggaaggacttcttggagtt
 P  I  A  A  I  R  S  P  L  S  S  D  Q  E  E  G  L  L  G  V 
cgatctatctcaactcaagtggtgcgtaaccgcggaggatacccttacgacgttcctgat
 R  S  I  S  T  Q  V  V  R  N  R  G  G  **Y  P  Y  D  V  P  D** 
tacgctggaggtatgacgtgtgaggtgaaggagaagggccgagttgggacgataaatcct
**Y  A**  G  G  M  T  C  E  V  K  E  K  G  R  V  G  T  I  N  P 
atttttacctgccagcctgcgggcgctcagtttgtatctatcgggatcaaagactgtatc
 I  F  T  C  Q  P  A  G  A  Q  F  V  S  I  G  I  K  D  C  I 
ggtatcgtgcatggaggacaagggtgcgtgatgttcgtgcgactcatcttctcacagcat
 G  I  V  H  G  G  Q  G  C  V  M  F  V  R  L  I  F  S  Q  H 
tacaaggagagctttgagctggcatcatcctctctccacgaggacggggccgtgttcggg
 Y  K  E  S  F  E  L  A  S  S  S  L  H  E  D  G  A  V  F  G 
gcctgtggccgtgttgaagaggcagttgatgtcttgctttcccgttatcccgacgtaaag
 A  C  G  R  V  E  E  A  V  D  V  L  L  S  R  Y  P  D  V  K 
gttgtaccaattattactacatgctccacggaaatcataggcgacgatgtggatggggtt
 V  V  P  I  I  T  T  C  S  T  E  I  I  G  D  D  V  D  G  V 
atcaagaagttgaatgagggtctgctgaaagagaagttccctgaccgagaagtccacctt
 I  K  K  L  N  E  G  L  L  K  E  K  F  P  D  R  E  V  H  L 
atagcgatgcatactcctagttttgtaggatccatgataagtggctacgatgttgctgtg
 I  A  M  H  T  P  S  F  V  G  S  M  I  S  G  Y  D  V  A  V 
cgtgatgtagtacgacacttcgccaagagagaagccccgaacgataaaataaatcttctt
 R  D  V  V  R  H  F  A  K  R  E  A  P  N  D  K  I  N  L  L 
acaggatgggtcaatcctggtgatgtgaaagaacttaaacatttactcggcgagatggat
 T  G  W  V  N  P  G  D  V  K  E  L  K  H  L  L  G  E  M  D 
attgaggcgaacgtactttttgaaattgaatcttttgactctcctatcttacctgacggc
 I  E  A  N  V  L  F  E  I  E  S  F  D  S  P  I  L  P  D  G 
tccgcggttagtcacggaaatacaactatagaggacctaattgacacaggtaacgcccga
 S  A  V  S  H  G  N  T  T  I  E  D  L  I  D  T  G  N  A  R 
gcgacgttcgctctaaacagatatgaaggaacgaaagcggcagagtatctacaaaagaaa
 A  T  F  A  L  N  R  Y  E  G  T  K  A  A  E  Y  L  Q  K  K 
tttgagattcctgccattataggcccaacgcccatagggattcgaaacacggacatattc
 F  E  I  P  A  I  I  G  P  T  P  I  G  I  R  N  T  D  I  F 
cttcagaatctgaagaaagctacagggaaaccaattccgcagagcttggcccacgagcgt
 L  Q  N  L  K  K  A  T  G  K  P  I  P  Q  S  L  A  H  E  R 
ggagtggctattgatgctttggcggacctgacacacatgtttctagcagaaaagagagtc
 G  V  A  I  D  A  L  A  D  L  T  H  M  F  L  A  E  K  R  V 
gctatctatggggcacccgatttagtgataggccttgcagagttctgcctagatctagag
 A  I  Y  G  A  P  D  L  V  I  G  L  A  E  F  C  L  D  L  E 
atgaagccggtccttttattacttggtgacgacaattccaaatacgtcgacgatcccagg
 M  K  P  V  L  L  L  L  G  D  D  N  S  K  Y  V  D  D  P  R 
attaaagccctacaagaaaacgtcgactacgggatggagattgttacgaacgcggacttc
 I  K  A  L  Q  E  N  V  D  Y  G  M  E  I  V  T  N  A  D  F 
tgggagctcgagaataggatcaagaatgagggcctcgaactcgacctgatactggggcac
 W  E  L  E  N  R  I  K  N  E  G  L  E  L  D  L  I  L  G  H 
tctaagggtaggtttatcagcatcgactacaatattccgatgttgagagtgggcttcccg
 S  K  G  R  F  I  S  I  D  Y  N  I  P  M  L  R  V  G  F  P 
acgtatgatcgagctggactttttaggtatcccaccgttggctacggaggggctatatgg
 T  Y  D  R  A  G  L  F  R  Y  P  T  V  G  Y  G  G  A  I  W 
ttagcggagcaaatggcgaacactttgttcgcagacatggaacacaagaaaaacaaggag
 L  A  E  Q  M  A  N  T  L  F  A  D  M  E  H  K  K  N  K  E 
tgggtattgaatgtatgg
 W  V  L  N  V  W

**SN130, pFAγ51-HA-AnfH:**

atggcaatggctgttttccgtcgcgaagggaggcgtctcctcccttcaatcgccgctcgc
 M  A  M  A  V  F  R  R  E  G  R  R  L  L  P  S  I  A  A  R 
ccaatcgctgctatccgatctcctctctcttctgaccaggaggaaggacttcttggagtt
 P  I  A  A  I  R  S  P  L  S  S  D  Q  E  E  G  L  L  G  V 
cgatctatctcaactcaagtggtgcgtaaccgcggaggatacccttacgacgttcctgat
 R  S  I  S  T  Q  V  V  R  N  R  G  G  **Y  P  Y  D  V  P  D** 
tacgctggaggtatgacgcgaaaagtggcgatttatggcaaaggcggtataggtaaatca
**Y  A**  G  G  M  T  R  K  V  A  I  Y  G  K  G  G  I  G  K  S 
acaacgacccaaaataccgctgctgctttggcctacttccatgataaaaaggttttcatc
 T  T  T  Q  N  T  A  A  A  L  A  Y  F  H  D  K  K  V  F  I 
catggctgcgaccctaaagcggacagtacacgattaatactaggcggaaagccccaggaa
 H  G  C  D  P  K  A  D  S  T  R  L  I  L  G  G  K  P  Q  E 
accctaatggatatgctacgtgataagggagctgagaaaattactaatgatgacgtaatc
 T  L  M  D  M  L  R  D  K  G  A  E  K  I  T  N  D  D  V  I 
aaaaaggggttcctcgacatacagtgcgttgagagtggtggacccgaaccaggcgtcggt
 K  K  G  F  L  D  I  Q  C  V  E  S  G  G  P  E  P  G  V  G 
tgtgcaggtaggggtgttataacggccatcgacctcatggaagagaatggagcgtacaca
 C  A  G  R  G  V  I  T  A  I  D  L  M  E  E  N  G  A  Y  T 
gacgacctagattttgtctttttcgatgtccttggtgatgtggtgtgcggcggttttgct
 D  D  L  D  F  V  F  F  D  V  L  G  D  V  V  C  G  G  F  A 
atgcccattagagatggaaaggcacaggaggtgtatattgttgcctccggggaaatgatg
 M  P  I  R  D  G  K  A  Q  E  V  Y  I  V  A  S  G  E  M  M 
gcgatttacgcggcaaacaatatctgcaagggtctagtgaaatatgcgaagcagtctgga
 A  I  Y  A  A  N  N  I  C  K  G  L  V  K  Y  A  K  Q  S  G 
gttagactggggggtataatttgtaacagtcgaaaagtggacggtgagagggaattcctc
 V  R  L  G  G  I  I  C  N  S  R  K  V  D  G  E  R  E  F  L 
gaggagtttaccgctgcaatagggacgaaaatgatacatttcgtgcccagagacaatatc
 E  E  F  T  A  A  I  G  T  K  M  I  H  F  V  P  R  D  N  I 
gtgcaaaaagctgaattcaataaaaagaccgtgacggagttcgctccagaggaaaaccaa
 V  Q  K  A  E  F  N  K  K  T  V  T  E  F  A  P  E  E  N  Q 
gctaaagaatatggagaactcgcccgtaaaatcatcgagaatgatgagttcgttatccca
 A  K  E  Y  G  E  L  A  R  K  I  I  E  N  D  E  F  V  I  P 
aagcctctcactatggaccagctagaggacatggtagtgaagtatgggatagccgat
 K  P  L  T  M  D  Q  L  E  D  M  V  V  K  Y  G  I  A  D

**SN131, pFAγ51-HA-AnfG:**

atggcaatggctgttttccgtcgcgaagggaggcgtctcctcccttcaatcgccgctcgc
 M  A  M  A  V  F  R  R  E  G  R  R  L  L  P  S  I  A  A  R 
ccaatcgctgctatccgatctcctctctcttctgaccaggaggaaggacttcttggagtt
 P  I  A  A  I  R  S  P  L  S  S  D  Q  E  E  G  L  L  G  V 
cgatctatctcaactcaagtggtgcgtaaccgcggaggatacccttacgacgttcctgat
 R  S  I  S  T  Q  V  V  R  N  R  G  G  **Y  P  Y  D  V  P  D** 
tacgctggaggtatgtccacggcctccgccgctgccgtagtcaaacagaaagtagaggcg
**Y  A**  G  G  M  S  T  A  S  A  A  A  V  V  K  Q  K  V  E  A 
cccgtacacccgatggatgcgagaatcgacgaacttacagattacataatgaagaactgc
 P  V  H  P  M  D  A  R  I  D  E  L  T  D  Y  I  M  K  N  C 
ctctggcagttccacagcaggagttgggacagggagaggcagaatgccgaaattctcaag
 L  W  Q  F  H  S  R  S  W  D  R  E  R  Q  N  A  E  I  L  K 
aaaacgaaagaattactttgtggcgagcctgtagatctatcaacatcacacgaccgatgc
 K  T  K  E  L  L  C  G  E  P  V  D  L  S  T  S  H  D  R  C 
tattgggttgacgctgtctgcctagcggatgactaccgtgagcactacccgtggataaac
 Y  W  V  D  A  V  C  L  A  D  D  Y  R  E  H  Y  P  W  I  N 
agtatgtccaaggaggagataggaagtctaatgcaaggattaaaggaccgtatggactac
 S  M  S  K  E  E  I  G  S  L  M  Q  G  L  K  D  R  M  D  Y 
ttgaccataacggggagcctaaatgaggagttatctgacaagcattac
 L  T  I  T  G  S  L  N  E  E  L  S  D  K  H  Y

**SN152, HA-AnfK:**

atgtacccttacgacgttcctgattacgctggaggtatgacgtgtgaggtgaaggagaag
 M  **Y  P  Y  D  V  P  D  Y  A**  G  G  M  T  C  E  V  K  E  K 
ggccgagttgggacgataaatcctatttttacctgccagcctgcgggcgctcagtttgta
 G  R  V  G  T  I  N  P  I  F  T  C  Q  P  A  G  A  Q  F  V 
tctatcgggatcaaagactgtatcggtatcgtgcatggaggacaagggtgcgtgatgttc
 S  I  G  I  K  D  C  I  G  I  V  H  G  G  Q  G  C  V  M  F 
gtgcgactcatcttctcacagcattacaaggagagctttgagctggcatcatcctctctc
 V  R  L  I  F  S  Q  H  Y  K  E  S  F  E  L  A  S  S  S  L 
cacgaggacggggccgtgttcggggcctgtggccgtgttgaagaggcagttgatgtcttg
 H  E  D  G  A  V  F  G  A  C  G  R  V  E  E  A  V  D  V  L 
ctttcccgttatcccgacgtaaaggttgtaccaattattactacatgctccacggaaatc
 L  S  R  Y  P  D  V  K  V  V  P  I  I  T  T  C  S  T  E  I 
ataggcgacgatgtggatggggttatcaagaagttgaatgagggtctgctgaaagagaag
 I  G  D  D  V  D  G  V  I  K  K  L  N  E  G  L  L  K  E  K 
ttccctgaccgagaagtccaccttatagcgatgcatactcctagttttgtaggatccatg
 F  P  D  R  E  V  H  L  I  A  M  H  T  P  S  F  V  G  S  M 
ataagtggctacgatgttgctgtgcgtgatgtagtacgacacttcgccaagagagaagcc
 I  S  G  Y  D  V  A  V  R  D  V  V  R  H  F  A  K  R  E  A 
ccgaacgataaaataaatcttcttacaggatgggtcaatcctggtgatgtgaaagaactt
 P  N  D  K  I  N  L  L  T  G  W  V  N  P  G  D  V  K  E  L 
aaacatttactcggcgagatggatattgaggcgaacgtactttttgaaattgaatctttt
 K  H  L  L  G  E  M  D  I  E  A  N  V  L  F  E  I  E  S  F 
gactctcctatcttacctgacggctccgcggttagtcacggaaatacaactatagaggac
 D  S  P  I  L  P  D  G  S  A  V  S  H  G  N  T  T  I  E  D 
ctaattgacacaggtaacgcccgagcgacgttcgctctaaacagatatgaaggaacgaaa
 L  I  D  T  G  N  A  R  A  T  F  A  L  N  R  Y  E  G  T  K 
gcggcagagtatctacaaaagaaatttgagattcctgccattataggcccaacgcccata
 A  A  E  Y  L  Q  K  K  F  E  I  P  A  I  I  G  P  T  P  I 
gggattcgaaacacggacatattccttcagaatctgaagaaagctacagggaaaccaatt
 G  I  R  N  T  D  I  F  L  Q  N  L  K  K  A  T  G  K  P  I 
ccgcagagcttggcccacgagcgtggagtggctattgatgctttggcggacctgacacac
 P  Q  S  L  A  H  E  R  G  V  A  I  D  A  L  A  D  L  T  H 
atgtttctagcagaaaagagagtcgctatctatggggcacccgatttagtgataggcctt
 M  F  L  A  E  K  R  V  A  I  Y  G  A  P  D  L  V  I  G  L 
gcagagttctgcctagatctagagatgaagccggtccttttattacttggtgacgacaat
 A  E  F  C  L  D  L  E  M  K  P  V  L  L  L  L  G  D  D  N 
tccaaatacgtcgacgatcccaggattaaagccctacaagaaaacgtcgactacgggatg
 S  K  Y  V  D  D  P  R  I  K  A  L  Q  E  N  V  D  Y  G  M 
gagattgttacgaacgcggacttctgggagctcgagaataggatcaagaatgagggcctc
 E  I  V  T  N  A  D  F  W  E  L  E  N  R  I  K  N  E  G  L 
gaactcgacctgatactggggcactctaagggtaggtttatcagcatcgactacaatatt
 E  L  D  L  I  L  G  H  S  K  G  R  F  I  S  I  D  Y  N  I 
ccgatgttgagagtgggcttcccgacgtatgatcgagctggactttttaggtatcccacc
 P  M  L  R  V  G  F  P  T  Y  D  R  A  G  L  F  R  Y  P  T 
gttggctacggaggggctatatggttagcggagcaaatggcgaacactttgttcgcagac
 V  G  Y  G  G  A  I  W  L  A  E  Q  M  A  N  T  L  F  A  D 
atggaacacaagaaaaacaaggagtgggtattgaatgtatgg
 M  E  H  K  K  N  K  E  W  V  L  N  V  W

**SN153, HA-AnfH:**

atgtacccttacgacgttcctgattacgctggaggtatgacgcgaaaagtggcgatttat
 M  **Y  P  Y  D  V  P  D  Y**  **A**  G  G  M  T  R  K  V  A  I  Y 
ggcaaaggcggtataggtaaatcaacaacgacccaaaataccgctgctgctttggcctac
 G  K  G  G  I  G  K  S  T  T  T  Q  N  T  A  A  A  L  A  Y 
ttccatgataaaaaggttttcatccatggctgcgaccctaaagcggacagtacacgatta
 F  H  D  K  K  V  F  I  H  G  C  D  P  K  A  D  S  T  R  L 
atactaggcggaaagccccaggaaaccctaatggatatgctacgtgataagggagctgag
 I  L  G  G  K  P  Q  E  T  L  M  D  M  L  R  D  K  G  A  E 
aaaattactaatgatgacgtaatcaaaaaggggttcctcgacatacagtgcgttgagagt
 K  I  T  N  D  D  V  I  K  K  G  F  L  D  I  Q  C  V  E  S 
ggtggacccgaaccaggcgtcggttgtgcaggtaggggtgttataacggccatcgacctc
 G  G  P  E  P  G  V  G  C  A  G  R  G  V  I  T  A  I  D  L 
atggaagagaatggagcgtacacagacgacctagattttgtctttttcgatgtccttggt
 M  E  E  N  G  A  Y  T  D  D  L  D  F  V  F  F  D  V  L  G 
gatgtggtgtgcggcggttttgctatgcccattagagatggaaaggcacaggaggtgtat
 D  V  V  C  G  G  F  A  M  P  I  R  D  G  K  A  Q  E  V  Y 
attgttgcctccggggaaatgatggcgatttacgcggcaaacaatatctgcaagggtcta
 I  V  A  S  G  E  M  M  A  I  Y  A  A  N  N  I  C  K  G  L 
gtgaaatatgcgaagcagtctggagttagactggggggtataatttgtaacagtcgaaaa
 V  K  Y  A  K  Q  S  G  V  R  L  G  G  I  I  C  N  S  R  K 
gtggacggtgagagggaattcctcgaggagtttaccgctgcaatagggacgaaaatgata
 V  D  G  E  R  E  F  L  E  E  F  T  A  A  I  G  T  K  M  I 
catttcgtgcccagagacaatatcgtgcaaaaagctgaattcaataaaaagaccgtgacg
 H  F  V  P  R  D  N  I  V  Q  K  A  E  F  N  K  K  T  V  T 
gagttcgctccagaggaaaaccaagctaaagaatatggagaactcgcccgtaaaatcatc
 E  F  A  P  E  E  N  Q  A  K  E  Y  G  E  L  A  R  K  I  I 
gagaatgatgagttcgttatcccaaagcctctcactatggaccagctagaggacatggta
 E  N  D  E  F  V  I  P  K  P  L  T  M  D  Q  L  E  D  M  V 
gtgaagtatgggatagccgat
 V  K  Y  G  I  A  D

**SN154, HA-AnfG:**

atgtacccttacgacgttcctgattacgctggaggtatgtccacggcctccgccgctgcc
 M  **Y  P  Y  D  V  P  D  Y  A**  G  G  M  S  T  A  S  A  A  A 
gtagtcaaacagaaagtagaggcgcccgtacacccgatggatgcgagaatcgacgaactt
 V  V  K  Q  K  V  E  A  P  V  H  P  M  D  A  R  I  D  E  L 
acagattacataatgaagaactgcctctggcagttccacagcaggagttgggacagggag
 T  D  Y  I  M  K  N  C  L  W  Q  F  H  S  R  S  W  D  R  E 
aggcagaatgccgaaattctcaagaaaacgaaagaattactttgtggcgagcctgtagat
 R  Q  N  A  E  I  L  K  K  T  K  E  L  L  C  G  E  P  V  D 
ctatcaacatcacacgaccgatgctattgggttgacgctgtctgcctagcggatgactac
 L  S  T  S  H  D  R  C  Y  W  V  D  A  V  C  L  A  D  D  Y 
cgtgagcactacccgtggataaacagtatgtccaaggaggagataggaagtctaatgcaa
 R  E  H  Y  P  W  I  N  S  M  S  K  E  E  I  G  S  L  M  Q 
ggattaaaggaccgtatggactacttgaccataacggggagcctaaatgaggagttatct
 G  L  K  D  R  M  D  Y  L  T  I  T  G  S  L  N  E  E  L  S 
gacaagcattac
 D  K  H  Y

**SN155, alaFAγ51-HA-AnfK:**

atggcaatggctgttttccgtcgcgaagcggcggctctcctcccttcaatcgccgctcgc
 M  A  M  A  V  F  R  R  E  A  A  A  L  L  P  S  I  A  A  R 
ccaatcgctgctgctgcagctgccgcttcttctgaccaggaggaaggacttcttgctgcc
 P  I  A  A  A  A  A  A  A  S  S  D  Q  E  E  G  L  L  A  A 
gcagcggctgcggccgctgtggtgcgtaaccgcggaggttacccttacgacgttcctgat
 A  A  A  A  A  A  V  V  R  N  R  G  G  **Y  P  Y  D  V  P  D** 
tacgctggaggtatgacgtgtgaggtgaaggagaagggccgagttgggacgataaatcct
 **Y  A**  G  G  M  T  C  E  V  K  E  K  G  R  V  G  T  I  N  P 
atttttacctgccagcctgcgggcgctcagtttgtatctatcgggatcaaagactgtatc
 I  F  T  C  Q  P  A  G  A  Q  F  V  S  I  G  I  K  D  C  I 
ggtatcgtgcatggaggacaagggtgcgtgatgttcgtgcgactcatcttctcacagcat
 G  I  V  H  G  G  Q  G  C  V  M  F  V  R  L  I  F  S  Q  H 
tacaaggagagctttgagctggcatcatcctctctccacgaggacggggccgtgttcggg
 Y  K  E  S  F  E  L  A  S  S  S  L  H  E  D  G  A  V  F  G 
gcctgtggccgtgttgaagaggcagttgatgtcttgctttcccgttatcccgacgtaaag
 A  C  G  R  V  E  E  A  V  D  V  L  L  S  R  Y  P  D  V  K 
gttgtaccaattattactacatgctccacggaaatcataggcgacgatgtggatggggtt
 V  V  P  I  I  T  T  C  S  T  E  I  I  G  D  D  V  D  G  V 
atcaagaagttgaatgagggtctgctgaaagagaagttccctgaccgagaagtccacctt
 I  K  K  L  N  E  G  L  L  K  E  K  F  P  D  R  E  V  H  L 
atagcgatgcatactcctagttttgtaggatccatgataagtggctacgatgttgctgtg
 I  A  M  H  T  P  S  F  V  G  S  M  I  S  G  Y  D  V  A  V 
cgtgatgtagtacgacacttcgccaagagagaagccccgaacgataaaataaatcttctt
 R  D  V  V  R  H  F  A  K  R  E  A  P  N  D  K  I  N  L  L 
acaggatgggtcaatcctggtgatgtgaaagaacttaaacatttactcggcgagatggat
 T  G  W  V  N  P  G  D  V  K  E  L  K  H  L  L  G  E  M  D 
attgaggcgaacgtactttttgaaattgaatcttttgactctcctatcttacctgacggc
 I  E  A  N  V  L  F  E  I  E  S  F  D  S  P  I  L  P  D  G 
tccgcggttagtcacggaaatacaactatagaggacctaattgacacaggtaacgcccga
 S  A  V  S  H  G  N  T  T  I  E  D  L  I  D  T  G  N  A  R 
gcgacgttcgctctaaacagatatgaaggaacgaaagcggcagagtatctacaaaagaaa
 A  T  F  A  L  N  R  Y  E  G  T  K  A  A  E  Y  L  Q  K  K 
tttgagattcctgccattataggcccaacgcccatagggattcgaaacacggacatattc
 F  E  I  P  A  I  I  G  P  T  P  I  G  I  R  N  T  D  I  F 
cttcagaatctgaagaaagctacagggaaaccaattccgcagagcttggcccacgagcgt
 L  Q  N  L  K  K  A  T  G  K  P  I  P  Q  S  L  A  H  E  R 
ggagtggctattgatgctttggcggacctgacacacatgtttctagcagaaaagagagtc
 G  V  A  I  D  A  L  A  D  L  T  H  M  F  L  A  E  K  R  V 
gctatctatggggcacccgatttagtgataggccttgcagagttctgcctagatctagag
 A  I  Y  G  A  P  D  L  V  I  G  L  A  E  F  C  L  D  L  E 
atgaagccggtccttttattacttggtgacgacaattccaaatacgtcgacgatcccagg
 M  K  P  V  L  L  L  L  G  D  D  N  S  K  Y  V  D  D  P  R 
attaaagccctacaagaaaacgtcgactacgggatggagattgttacgaacgcggacttc
 I  K  A  L  Q  E  N  V  D  Y  G  M  E  I  V  T  N  A  D  F 
tgggagctcgagaataggatcaagaatgagggcctcgaactcgacctgatactggggcac
 W  E  L  E  N  R  I  K  N  E  G  L  E  L  D  L  I  L  G  H 
tctaagggtaggtttatcagcatcgactacaatattccgatgttgagagtgggcttcccg
 S  K  G  R  F  I  S  I  D  Y  N  I  P  M  L  R  V  G  F  P 
acgtatgatcgagctggactttttaggtatcccaccgttggctacggaggggctatatgg
 T  Y  D  R  A  G  L  F  R  Y  P  T  V  G  Y  G  G  A  I  W 
ttagcggagcaaatggcgaacactttgttcgcagacatggaacacaagaaaaacaaggag
 L  A  E  Q  M  A  N  T  L  F  A  D  M  E  H  K  K  N  K  E 
tgggtattgaatgtatgg
 W  V  L  N  V  W

**SN156, alaFAγ51-HA-AnfH:**

atggcaatggctgttttccgtcgcgaagcggcggctctcctcccttcaatcgccgctcgc
 M  A  M  A  V  F  R  R  E  A  A  A  L  L  P  S  I  A  A  R 
ccaatcgctgctgctgcagctgccgcttcttctgaccaggaggaaggacttcttgctgcc
 P  I  A  A  A  A  A  A  A  S  S  D  Q  E  E  G  L  L  A  A 
gcagcggctgcggccgctgtggtgcgtaaccgcggaggttacccttacgacgttcctgat
 A  A  A  A  A  A  V  V  R  N  R  G  G  **Y  P  Y  D  V  P  D** 
tacgctggaggtatgacgcgaaaagtggcgatttatggcaaaggcggtataggtaaatca
 **Y  A**  G  G  M  T  R  K  V  A  I  Y  G  K  G  G  I  G  K  S 
acaacgacccaaaataccgctgctgctttggcctacttccatgataaaaaggttttcatc
 T  T  T  Q  N  T  A  A  A  L  A  Y  F  H  D  K  K  V  F  I 
catggctgcgaccctaaagcggacagtacacgattaatactaggcggaaagccccaggaa
 H  G  C  D  P  K  A  D  S  T  R  L  I  L  G  G  K  P  Q  E 
accctaatggatatgctacgtgataagggagctgagaaaattactaatgatgacgtaatc
 T  L  M  D  M  L  R  D  K  G  A  E  K  I  T  N  D  D  V  I 
aaaaaggggttcctcgacatacagtgcgttgagagtggtggacccgaaccaggcgtcggt
 K  K  G  F  L  D  I  Q  C  V  E  S  G  G  P  E  P  G  V  G 
tgtgcaggtaggggtgttataacggccatcgacctcatggaagagaatggagcgtacaca
 C  A  G  R  G  V  I  T  A  I  D  L  M  E  E  N  G  A  Y  T 
gacgacctagattttgtctttttcgatgtccttggtgatgtggtgtgcggcggttttgct
 D  D  L  D  F  V  F  F  D  V  L  G  D  V  V  C  G  G  F  A 
atgcccattagagatggaaaggcacaggaggtgtatattgttgcctccggggaaatgatg
 M  P  I  R  D  G  K  A  Q  E  V  Y  I  V  A  S  G  E  M  M 
gcgatttacgcggcaaacaatatctgcaagggtctagtgaaatatgcgaagcagtctgga
 A  I  Y  A  A  N  N  I  C  K  G  L  V  K  Y  A  K  Q  S  G 
gttagactggggggtataatttgtaacagtcgaaaagtggacggtgagagggaattcctc
 V  R  L  G  G  I  I  C  N  S  R  K  V  D  G  E  R  E  F  L 
gaggagtttaccgctgcaatagggacgaaaatgatacatttcgtgcccagagacaatatc
 E  E  F  T  A  A  I  G  T  K  M  I  H  F  V  P  R  D  N  I 
gtgcaaaaagctgaattcaataaaaagaccgtgacggagttcgctccagaggaaaaccaa
 V  Q  K  A  E  F  N  K  K  T  V  T  E  F  A  P  E  E  N  Q 
gctaaagaatatggagaactcgcccgtaaaatcatcgagaatgatgagttcgttatccca
 A  K  E  Y  G  E  L  A  R  K  I  I  E  N  D  E  F  V  I  P 
aagcctctcactatggaccagctagaggacatggtagtgaagtatgggatagccgat
 K  P  L  T  M  D  Q  L  E  D  M  V  V  K  Y  G  I  A  D

**SN157, alaFAγ51-HA-AnfG:**

atggcaatggctgttttccgtcgcgaagcggcggctctcctcccttcaatcgccgctcgc
 M  A  M  A  V  F  R  R  E  A  A  A  L  L  P  S  I  A  A  R 
ccaatcgctgctgctgcagctgccgcttcttctgaccaggaggaaggacttcttgctgcc
 P  I  A  A  A  A  A  A  A  S  S  D  Q  E  E  G  L  L  A  A 
gcagcggctgcggccgctgtggtgcgtaaccgcggaggttacccttacgacgttcctgat
 A  A  A  A  A  A  V  V  R  N  R  G  G  **Y  P  Y  D  V  P  D** 
tacgctggaggtatgtccacggcctccgccgctgccgtagtcaaacagaaagtagaggcg
**Y  A**  G  G  M  S  T  A  S  A  A  A  V  V  K  Q  K  V  E  A 
cccgtacacccgatggatgcgagaatcgacgaacttacagattacataatgaagaactgc
 P  V  H  P  M  D  A  R  I  D  E  L  T  D  Y  I  M  K  N  C 
ctctggcagttccacagcaggagttgggacagggagaggcagaatgccgaaattctcaag
 L  W  Q  F  H  S  R  S  W  D  R  E  R  Q  N  A  E  I  L  K 
aaaacgaaagaattactttgtggcgagcctgtagatctatcaacatcacacgaccgatgc
 K  T  K  E  L  L  C  G  E  P  V  D  L  S  T  S  H  D  R  C 
tattgggttgacgctgtctgcctagcggatgactaccgtgagcactacccgtggataaac
 Y  W  V  D  A  V  C  L  A  D  D  Y  R  E  H  Y  P  W  I  N 
agtatgtccaaggaggagataggaagtctaatgcaaggattaaaggaccgtatggactac
 S  M  S  K  E  E  I  G  S  L  M  Q  G  L  K  D  R  M  D  Y 
ttgaccataacggggagcctaaatgaggagttatctgacaagcattac
 L  T  I  T  G  S  L  N  E  E  L  S  D  K  H  Y

**SN158, alaFAγ51-HA-AnfD:**

atggcaatggctgttttccgtcgcgaagcggcggctctcctcccttcaatcgccgctcgc
 M  A  M  A  V  F  R  R  E  A  A  A  L  L  P  S  I  A  A  R 
ccaatcgctgctgctgcagctgccgcttcttctgaccaggaggaaggacttcttgctgcc
 P  I  A  A  A  A  A  A  A  S  S  D  Q  E  E  G  L  L  A  A 
gcagcggctgcggccgctgtggtgcgtaaccgcggaggttacccttacgacgttcctgat
 A  A  A  A  A  A  V  V  R  N  R  G  G  **Y  P  Y  D  V  P  D** 
tacgctggaggtatgcctcatcatgagttcgagtgttccaaagtaatcccggagagaaag
**Y  A**  G  G  M  P  H  H  E  F  E  C  S  K  V  I  P  E  R  K 
aagcatgccgttataaaaggaaagggcgagacactagcggacgcgcttcctcaaggatat
 K  H  A  V  I  K  G  K  G  E  T  L  A  D  A  L  P  Q  G  Y 
ctaaacacaatacctgggtcaatttccgaaagaggttgtgcttattgcggtgcaaagcac
 L  N  T  I  P  G  S  I  S  E  R  G  C  A  Y  C  G  A  K  H 
gttattggaaccccaatgaaagatgttatacacatttcccacggaccagttggctgtact
 V  I  G  T  P  M  K  D  V  I  H  I  S  H  G  P  V  G  C  T 
tacgatacatggcagacgaaacgttacatatccgacaatgacaatttccagctaaagtat
 Y  D  T  W  Q  T  K  R  Y  I  S  D  N  D  N  F  Q  L  K  Y 
acttatgcgacggacgtaaaagaaaaacacatagtctttggagctgaaaaattactgaaa
 T  Y  A  T  D  V  K  E  K  H  I  V  F  G  A  E  K  L  L  K 
caaaacatcattgaggcgtttaaggcattcccgcaaatcaagcgaatgactatctaccag
 Q  N  I  I  E  A  F  K  A  F  P  Q  I  K  R  M  T  I  Y  Q 
acttgtgcgactgctttgatcggagatgatattaatgccatcgcggaagaggtaatggag
 T  C  A  T  A  L  I  G  D  D  I  N  A  I  A  E  E  V  M  E 
gagatgcctgaagttgacatatttgtttgcaactctcccggattcgctggaccatctcag
 E  M  P  E  V  D  I  F  V  C  N  S  P  G  F  A  G  P  S  Q 
agtggcgggcatcacaagatcaatattgcctggattaatcagaaggtcggcacagtagag
 S  G  G  H  H  K  I  N  I  A  W  I  N  Q  K  V  G  T  V  E 
cctgaaattaccggtgaccatgtcataaactatgtgggggaatataacatacaaggcgac
 P  E  I  T  G  D  H  V  I  N  Y  V  G  E  Y  N  I  Q  G  D 
caggaggttatggttgactactttaagagaatggggatccaggtcttgtccactttcacc
 Q  E  V  M  V  D  Y  F  K  R  M  G  I  Q  V  L  S  T  F  T 
ggcaacggaagttatgatgggttgagagctatgcaccgtgcgcacttgaatgtattagag
 G  N  G  S  Y  D  G  L  R  A  M  H  R  A  H  L  N  V  L  E 
tgtgctcgtagcgcagagtacatttgcaatgaactaagagttagatacggcatccctcga
 C  A  R  S  A  E  Y  I  C  N  E  L  R  V  R  Y  G  I  P  R 
cttgatatagacggttttggattcaaaccacttgcggactcactccgtaagattggaatg
 L  D  I  D  G  F  G  F  K  P  L  A  D  S  L  R  K  I  G  M 
ttctttgggatagaggaccgtgcaaaagccatcatcgacgaggaagtagctcgttggaaa
 F  F  G  I  E  D  R  A  K  A  I  I  D  E  E  V  A  R  W  K 
ccggaattggattggtataaggagcgactcatgggcaagaaggtgtgcctatggcctggt
 P  E  L  D  W  Y  K  E  R  L  M  G  K  K  V  C  L  W  P  G 
ggctccaaattgtggcattgggcgcacgtcatcgaggaagaaatgggattgaaggtcgtg
 G  S  K  L  W  H  W  A  H  V  I  E  E  E  M  G  L  K  V  V 
tccgtctacaccaagttcggacatcaaggcgacatggagaagggaatagcccgatgtggc
 S  V  Y  T  K  F  G  H  Q  G  D  M  E  K  G  I  A  R  C  G 
gaaggtactcttgcgatcgatgatccaaatgaactcgaaggtttagaagctcttgagatg
 E  G  T  L  A  I  D  D  P  N  E  L  E  G  L  E  A  L  E  M 
cttaaaccagatataattttgacgggaaagcgtccaggtgaggtggcaaaaaaagtccgt
 L  K  P  D  I  I  L  T  G  K  R  P  G  E  V  A  K  K  V  R 
gttccttacctcaatgcccacgcttaccacaacgggccttacaaaggttttgaaggttgg
 V  P  Y  L  N  A  H  A  Y  H  N  G  P  Y  K  G  F  E  G  W 
gtgcgttttgctagggatatttataatgctatctacagtccaatccatcagctgtccgga
 V  R  F  A  R  D  I  Y  N  A  I  Y  S  P  I  H  Q  L  S  G 
atcgatataactaaggataatgcccctgaatgggggaatggctttcgtacgcgacagatg
 I  D  I  T  K  D  N  A  P  E  W  G  N  G  F  R  T  R  Q  M 
ctcagtgacgggaatttgagcgacgctgtcaggaattcagaaacactgcgtcagtacaca
 L  S  D  G  N  L  S  D  A  V  R  N  S  E  T  L  R  Q  Y  T 
ggcggttatgactctgtatcaaaactgcgagaaagagagtatcccgcgtttgaacgtaag
 G  G  Y  D  S  V  S  K  L  R  E  R  E  Y  P  A  F  E  R  K 
gttggt V  G

**SN161, pFAγ51-HA-AnfD:**

atggcaatggctgttttccgtcgcgaagggaggcgtctcctcccttcaatcgccgctcgc
 M  A  M  A  V  F  R  R  E  G  R  R  L  L  P  S  I  A  A  R 
ccaatcgctgctatccgatctcctctctcttctgaccaggaggaaggacttcttggagtt
 P  I  A  A  I  R  S  P  L  S  S  D  Q  E  E  G  L  L  G  V 
cgatctatctcaactcaagtggtgcgtaaccgcggaggatacccttacgacgttcctgat
 R  S  I  S  T  Q  V  V  R  N  R  G  G  **Y  P  Y  D  V  P  D** 
tacgctggaggtatgcctcatcatgagttcgagtgttccaaagtaatcccggagagaaag
 **Y  A**  G  G  M  P  H  H  E  F  E  C  S  K  V  I  P  E  R  K 
aagcatgccgttataaaaggaaagggcgagacactagcggacgcgcttcctcaaggatat
 K  H  A  V  I  K  G  K  G  E  T  L  A  D  A  L  P  Q  G  Y 
ctaaacacaatacctgggtcaatttccgaaagaggttgtgcttattgcggtgcaaagcac
 L  N  T  I  P  G  S  I  S  E  R  G  C  A  Y  C  G  A  K  H 
gttattggaaccccaatgaaagatgttatacacatttcccacggaccagttggctgtact
 V  I  G  T  P  M  K  D  V  I  H  I  S  H  G  P  V  G  C  T 
tacgatacatggcagacgaaacgttacatatccgacaatgacaatttccagctaaagtat
 Y  D  T  W  Q  T  K  R  Y  I  S  D  N  D  N  F  Q  L  K  Y 
acttatgcgacggacgtaaaagaaaaacacatagtctttggagctgaaaaattactgaaa
 T  Y  A  T  D  V  K  E  K  H  I  V  F  G  A  E  K  L  L  K 
caaaacatcattgaggcgtttaaggcattcccgcaaatcaagcgaatgactatctaccag
 Q  N  I  I  E  A  F  K  A  F  P  Q  I  K  R  M  T  I  Y  Q 
acttgtgcgactgctttgatcggagatgatattaatgccatcgcggaagaggtaatggag
 T  C  A  T  A  L  I  G  D  D  I  N  A  I  A  E  E  V  M  E 
gagatgcctgaagttgacatatttgtttgcaactctcccggattcgctggaccatctcag
 E  M  P  E  V  D  I  F  V  C  N  S  P  G  F  A  G  P  S  Q 
agtggcgggcatcacaagatcaatattgcctggattaatcagaaggtcggcacagtagag
 S  G  G  H  H  K  I  N  I  A  W  I  N  Q  K  V  G  T  V  E 
cctgaaattaccggtgaccatgtcataaactatgtgggggaatataacatacaaggcgac
 P  E  I  T  G  D  H  V  I  N  Y  V  G  E  Y  N  I  Q  G  D 
caggaggttatggttgactactttaagagaatggggatccaggtcttgtccactttcacc
 Q  E  V  M  V  D  Y  F  K  R  M  G  I  Q  V  L  S  T  F  T 
ggcaacggaagttatgatgggttgagagctatgcaccgtgcgcacttgaatgtattagag
 G  N  G  S  Y  D  G  L  R  A  M  H  R  A  H  L  N  V  L  E 
tgtgctcgtagcgcagagtacatttgcaatgaactaagagttagatacggcatccctcga
 C  A  R  S  A  E  Y  I  C  N  E  L  R  V  R  Y  G  I  P  R 
cttgatatagacggttttggattcaaaccacttgcggactcactccgtaagattggaatg
 L  D  I  D  G  F  G  F  K  P  L  A  D  S  L  R  K  I  G  M 
ttctttgggatagaggaccgtgcaaaagccatcatcgacgaggaagtagctcgttggaaa
 F  F  G  I  E  D  R  A  K  A  I  I  D  E  E  V  A  R  W  K 
ccggaattggattggtataaggagcgactcatgggcaagaaggtgtgcctatggcctggt
 P  E  L  D  W  Y  K  E  R  L  M  G  K  K  V  C  L  W  P  G 
ggctccaaattgtggcattgggcgcacgtcatcgaggaagaaatgggattgaaggtcgtg
 G  S  K  L  W  H  W  A  H  V  I  E  E  E  M  G  L  K  V  V 
tccgtctacaccaagttcggacatcaaggcgacatggagaagggaatagcccgatgtggc
 S  V  Y  T  K  F  G  H  Q  G  D  M  E  K  G  I  A  R  C  G 
gaaggtactcttgcgatcgatgatccaaatgaactcgaaggtttagaagctcttgagatg
 E  G  T  L  A  I  D  D  P  N  E  L  E  G  L  E  A  L  E  M 
cttaaaccagatataattttgacgggaaagcgtccaggtgaggtggcaaaaaaagtccgt
 L  K  P  D  I  I  L  T  G  K  R  P  G  E  V  A  K  K  V  R 
gttccttacctcaatgcccacgcttaccacaacgggccttacaaaggttttgaaggttgg
 V  P  Y  L  N  A  H  A  Y  H  N  G  P  Y  K  G  F  E  G  W 
gtgcgttttgctagggatatttataatgctatctacagtccaatccatcagctgtccgga
 V  R  F  A  R  D  I  Y  N  A  I  Y  S  P  I  H  Q  L  S  G 
atcgatataactaaggataatgcccctgaatgggggaatggctttcgtacgcgacagatg
 I  D  I  T  K  D  N  A  P  E  W  G  N  G  F  R  T  R  Q  M 
ctcagtgacgggaatttgagcgacgctgtcaggaattcagaaacactgcgtcagtacaca
 L  S  D  G  N  L  S  D  A  V  R  N  S  E  T  L  R  Q  Y  T 
ggcggttatgactctgtatcaaaactgcgagaaagagagtatcccgcgtttgaacgtaag
 G  G  Y  D  S  V  S  K  L  R  E  R  E  Y  P  A  F  E  R  K 
gttggt
 V  G

**SN177, pFAγ51-AnfD-Twin Strep:**

atgatggcaatggctgttttccgtcgcgaagggaggcgtctcctcccttcaatcgccgct
 M  M  A  M  A  V  F  R  R  E  G  R  R  L  L  P  S  I  A  A 
cgcccaatcgctgctatccgatctcctctctcttctgaccaggaggaaggacttcttgga
 R  P  I  A  A  I  R  S  P  L  S  S  D  Q  E  E  G  L  L  G 
gttcgatctatctcaactcaagtggtgcgtaaccgcggaggtatgcctcatcatgagttc
 V  R  S  I  S  T  Q  V  V  R  N  R  G  G  M  P  H  H  E  F 
gagtgttccaaagtaatcccggagagaaagaagcatgccgttataaaaggaaagggcgag
 E  C  S  K  V  I  P  E  R  K  K  H  A  V  I  K  G  K  G  E 
acactagcggacgcgcttcctcaaggatatctaaacacaatacctgggtcaatttccgaa
 T  L  A  D  A  L  P  Q  G  Y  L  N  T  I  P  G  S  I  S  E 
agaggttgtgcttattgcggtgcaaagcacgttattggaaccccaatgaaagatgttata
 R  G  C  A  Y  C  G  A  K  H  V  I  G  T  P  M  K  D  V  I 
cacatttcccacggaccagttggctgtacttacgatacatggcagacgaaacgttacata
 H  I  S  H  G  P  V  G  C  T  Y  D  T  W  Q  T  K  R  Y  I 
tccgacaatgacaatttccagctaaagtatacttatgcgacggacgtaaaagaaaaacac
 S  D  N  D  N  F  Q  L  K  Y  T  Y  A  T  D  V  K  E  K  H 
atagtctttggagctgaaaaattactgaaacaaaacatcattgaggcgtttaaggcattc
 I  V  F  G  A  E  K  L  L  K  Q  N  I  I  E  A  F  K  A  F 
ccgcaaatcaagcgaatgactatctaccagacttgtgcgactgctttgatcggagatgat
 P  Q  I  K  R  M  T  I  Y  Q  T  C  A  T  A  L  I  G  D  D 
attaatgccatcgcggaagaggtaatggaggagatgcctgaagttgacatatttgtttgc
 I  N  A  I  A  E  E  V  M  E  E  M  P  E  V  D  I  F  V  C 
aactctcccggattcgctggaccatctcagagtggcgggcatcacaagatcaatattgcc
 N  S  P  G  F  A  G  P  S  Q  S  G  G  H  H  K  I  N  I  A 
tggattaatcagaaggtcggcacagtagagcctgaaattaccggtgaccatgtcataaac
 W  I  N  Q  K  V  G  T  V  E  P  E  I  T  G  D  H  V  I  N 
tatgtgggggaatataacatacaaggcgaccaggaggttatggttgactactttaagaga
 Y  V  G  E  Y  N  I  Q  G  D  Q  E  V  M  V  D  Y  F  K  R 
atggggatccaggtcttgtccactttcaccggcaacggaagttatgatgggttgagagct
 M  G  I  Q  V  L  S  T  F  T  G  N  G  S  Y  D  G  L  R  A 
atgcaccgtgcgcacttgaatgtattagagtgtgctcgtagcgcagagtacatttgcaat
 M  H  R  A  H  L  N  V  L  E  C  A  R  S  A  E  Y  I  C  N 
gaactaagagttagatacggcatccctcgacttgatatagacggttttggattcaaacca
 E  L  R  V  R  Y  G  I  P  R  L  D  I  D  G  F  G  F  K  P 
cttgcggactcactccgtaagattggaatgttctttgggatagaggaccgtgcaaaagcc
 L  A  D  S  L  R  K  I  G  M  F  F  G  I  E  D  R  A  K  A 
atcatcgacgaggaagtagctcgttggaaaccggaattggattggtataaggagcgactc
 I  I  D  E  E  V  A  R  W  K  P  E  L  D  W  Y  K  E  R  L 
atgggcaagaaggtgtgcctatggcctggtggctccaaattgtggcattgggcgcacgtc
 M  G  K  K  V  C  L  W  P  G  G  S  K  L  W  H  W  A  H  V 
atcgaggaagaaatgggattgaaggtcgtgtccgtctacaccaagttcggacatcaaggc
 I  E  E  E  M  G  L  K  V  V  S  V  Y  T  K  F  G  H  Q  G 
gacatggagaagggaatagcccgatgtggcgaaggtactcttgcgatcgatgatccaaat
 D  M  E  K  G  I  A  R  C  G  E  G  T  L  A  I  D  D  P  N 
gaactcgaaggtttagaagctcttgagatgcttaaaccagatataattttgacgggaaag
 E  L  E  G  L  E  A  L  E  M  L  K  P  D  I  I  L  T  G  K 
cgtccaggtgaggtggcaaaaaaagtccgtgttccttacctcaatgcccacgcttaccac
 R  P  G  E  V  A  K  K  V  R  V  P  Y  L  N  A  H  A  Y  H 
aacgggccttacaaaggttttgaaggttgggtgcgttttgctagggatatttataatgct
 N  G  P  Y  K  G  F  E  G  W  V  R  F  A  R  D  I  Y  N  A 
atctacagtccaatccatcagctgtccggaatcgatataactaaggataatgcccctgaa
 I  Y  S  P  I  H  Q  L  S  G  I  D  I  T  K  D  N  A  P  E 
tgggggaatggctttcgtacgcgacagatgctcagtgacgggaatttgagcgacgctgtc
 W  G  N  G  F  R  T  R  Q  M  L  S  D  G  N  L  S  D  A  V 
aggaattcagaaacactgcgtcagtacacaggcggttatgactctgtatcaaaactgcga
 R  N  S  E  T  L  R  Q  Y  T  G  G  Y  D  S  V  S  K  L  R 
gaaagagagtatcccgcgtttgaacgtaaggttggtggtggctcagcatggagtcatcct
 E  R  E  Y  P  A  F  E  R  K  V  G  G  G  **S  A  W  S  H  P** 
cagtttgagaaaggtggaggttcaggtggtggaagcggtggatctgcttggtcacatcca
 **Q  F  E  K  G  G  G  S  G  G  G  S  G  G  S  A  W  S  H  P** 
caatttgaaaaa
 **Q  F  E  K**

**SN195, CoxIV-Twin Strep-AnfK:**

atgctttcacttagacaatctattagatttttcaagccagctacaagaactttgtgttct
 M  L  S  L  R  Q  S  I  R  F  F  K  P  A  T  R  T  L  C  S 
tctagatatcttcttcagcaaaaaccttcagcatggagtcatcctcagtttgagaaaggt
 S  R  Y  L  L  Q  Q  K  P  **S  A  W  S  H  P  Q  F  E  K  G** 
ggaggttcaggtggtggaagcggtggatctgcttggtcacatccacaatttgaaaaagga
 **G  G  S  G  G  G  S  G  G  S  A  W  S  H  P  Q  F  E**  **K** G 
ggtatgacgtgtgaggtgaaggagaagggccgagttgggacgataaatcctatttttacc
 G  M  T  C  E  V  K  E  K  G  R  V  G  T  I  N  P  I  F  T 
tgccagcctgcgggcgctcagtttgtatctatcgggatcaaagactgtatcggtatcgtg
 C  Q  P  A  G  A  Q  F  V  S  I  G  I  K  D  C  I  G  I  V 
catggaggacaagggtgcgtgatgttcgtgcgactcatcttctcacagcattacaaggag
 H  G  G  Q  G  C  V  M  F  V  R  L  I  F  S  Q  H  Y  K  E 
agctttgagctggcatcatcctctctccacgaggacggggccgtgttcggggcctgtggc
 S  F  E  L  A  S  S  S  L  H  E  D  G  A  V  F  G  A  C  G 
cgtgttgaagaggcagttgatgtcttgctttcccgttatcccgacgtaaaggttgtacca
 R  V  E  E  A  V  D  V  L  L  S  R  Y  P  D  V  K  V  V  P 
attattactacatgctccacggaaatcataggcgacgatgtggatggggttatcaagaag
 I  I  T  T  C  S  T  E  I  I  G  D  D  V  D  G  V  I  K  K 
ttgaatgagggtctgctgaaagagaagttccctgaccgagaagtccaccttatagcgatg
 L  N  E  G  L  L  K  E  K  F  P  D  R  E  V  H  L  I  A  M 
catactcctagttttgtaggatccatgataagtggctacgatgttgctgtgcgtgatgta
 H  T  P  S  F  V  G  S  M  I  S  G  Y  D  V  A  V  R  D  V 
gtacgacacttcgccaagagagaagccccgaacgataaaataaatcttcttacaggatgg
 V  R  H  F  A  K  R  E  A  P  N  D  K  I  N  L  L  T  G  W 
gtcaatcctggtgatgtgaaagaacttaaacatttactcggcgagatggatattgaggcg
 V  N  P  G  D  V  K  E  L  K  H  L  L  G  E  M  D  I  E  A 
aacgtactttttgaaattgaatcttttgactctcctatcttacctgacggctccgcggtt
 N  V  L  F  E  I  E  S  F  D  S  P  I  L  P  D  G  S  A  V 
agtcacggaaatacaactatagaggacctaattgacacaggtaacgcccgagcgacgttc
 S  H  G  N  T  T  I  E  D  L  I  D  T  G  N  A  R  A  T  F 
gctctaaacagatatgaaggaacgaaagcggcagagtatctacaaaagaaatttgagatt
 A  L  N  R  Y  E  G  T  K  A  A  E  Y  L  Q  K  K  F  E  I 
cctgccattataggcccaacgcccatagggattcgaaacacggacatattccttcagaat
 P  A  I  I  G  P  T  P  I  G  I  R  N  T  D  I  F  L  Q  N 
ctgaagaaagctacagggaaaccaattccgcagagcttggcccacgagcgtggagtggct
 L  K  K  A  T  G  K  P  I  P  Q  S  L  A  H  E  R  G  V  A 
attgatgctttggcggacctgacacacatgtttctagcagaaaagagagtcgctatctat
 I  D  A  L  A  D  L  T  H  M  F  L  A  E  K  R  V  A  I  Y 
ggggcacccgatttagtgataggccttgcagagttctgcctagatctagagatgaagccg
 G  A  P  D  L  V  I  G  L  A  E  F  C  L  D  L  E  M  K  P 
gtccttttattacttggtgacgacaattccaaatacgtcgacgatcccaggattaaagcc
 V  L  L  L  L  G  D  D  N  S  K  Y  V  D  D  P  R  I  K  A 
ctacaagaaaacgtcgactacgggatggagattgttacgaacgcggacttctgggagctc
 L  Q  E  N  V  D  Y  G  M  E  I  V  T  N  A  D  F  W  E  L 
gagaataggatcaagaatgagggcctcgaactcgacctgatactggggcactctaagggt
 E  N  R  I  K  N  E  G  L  E  L  D  L  I  L  G  H  S  K  G 
aggtttatcagcatcgactacaatattccgatgttgagagtgggcttcccgacgtatgat
 R  F  I  S  I  D  Y  N  I  P  M  L  R  V  G  F  P  T  Y  D 
cgagctggactttttaggtatcccaccgttggctacggaggggctatatggttagcggag
 R  A  G  L  F  R  Y  P  T  V  G  Y  G  G  A  I  W  L  A  E 
caaatggcgaacactttgttcgcagacatggaacacaagaaaaacaaggagtgggtattg
 Q  M  A  N  T  L  F  A  D  M  E  H  K  K  N  K  E  W  V  L 
aatgtatgg
 N  V  W

**SN227, pFAγ51-AnfD-HA:**

atgatggcaatggctgttttccgtcgcgaagggaggcgtctcctcccttcaatcgccgct
 M  M  A  M  A  V  F  R  R  E  G  R  R  L  L  P  S  I  A  A 
cgcccaatcgctgctatccgatctcctctctcttctgaccaggaggaaggacttcttgga
 R  P  I  A  A  I  R  S  P  L  S  S  D  Q  E  E  G  L  L  G 
gttcgatctatctcaactcaagtggtgcgtaaccgcggaggtatgcctcatcatgagttc
 V  R  S  I  S  T  Q  V  V  R  N  R  G  G  M  P  H  H  E  F 
gagtgttccaaagtaatcccggagagaaagaagcatgccgttataaaaggaaagggcgag
 E  C  S  K  V  I  P  E  R  K  K  H  A  V  I  K  G  K  G  E 
acactagcggacgcgcttcctcaaggatatctaaacacaatacctgggtcaatttccgaa
 T  L  A  D  A  L  P  Q  G  Y  L  N  T  I  P  G  S  I  S  E 
agaggttgtgcttattgcggtgcaaagcacgttattggaaccccaatgaaagatgttata
 R  G  C  A  Y  C  G  A  K  H  V  I  G  T  P  M  K  D  V  I 
cacatttcccacggaccagttggctgtacttacgatacatggcagacgaaacgttacata
 H  I  S  H  G  P  V  G  C  T  Y  D  T  W  Q  T  K  R  Y  I 
tccgacaatgacaatttccagctaaagtatacttatgcgacggacgtaaaagaaaaacac
 S  D  N  D  N  F  Q  L  K  Y  T  Y  A  T  D  V  K  E  K  H 
atagtctttggagctgaaaaattactgaaacaaaacatcattgaggcgtttaaggcattc
 I  V  F  G  A  E  K  L  L  K  Q  N  I  I  E  A  F  K  A  F 
ccgcaaatcaagcgaatgactatctaccagacttgtgcgactgctttgatcggagatgat
 P  Q  I  K  R  M  T  I  Y  Q  T  C  A  T  A  L  I  G  D  D 
attaatgccatcgcggaagaggtaatggaggagatgcctgaagttgacatatttgtttgc
 I  N  A  I  A  E  E  V  M  E  E  M  P  E  V  D  I  F  V  C 
aactctcccggattcgctggaccatctcagagtggcgggcatcacaagatcaatattgcc
 N  S  P  G  F  A  G  P  S  Q  S  G  G  H  H  K  I  N  I  A 
tggattaatcagaaggtcggcacagtagagcctgaaattaccggtgaccatgtcataaac
 W  I  N  Q  K  V  G  T  V  E  P  E  I  T  G  D  H  V  I  N 
tatgtgggggaatataacatacaaggcgaccaggaggttatggttgactactttaagaga
 Y  V  G  E  Y  N  I  Q  G  D  Q  E  V  M  V  D  Y  F  K  R 
atggggatccaggtcttgtccactttcaccggcaacggaagttatgatgggttgagagct
 M  G  I  Q  V  L  S  T  F  T  G  N  G  S  Y  D  G  L  R  A 
atgcaccgtgcgcacttgaatgtattagagtgtgctcgtagcgcagagtacatttgcaat
 M  H  R  A  H  L  N  V  L  E  C  A  R  S  A  E  Y  I  C  N 
gaactaagagttagatacggcatccctcgacttgatatagacggttttggattcaaacca
 E  L  R  V  R  Y  G  I  P  R  L  D  I  D  G  F  G  F  K  P 
cttgcggactcactccgtaagattggaatgttctttgggatagaggaccgtgcaaaagcc
 L  A  D  S  L  R  K  I  G  M  F  F  G  I  E  D  R  A  K  A 
atcatcgacgaggaagtagctcgttggaaaccggaattggattggtataaggagcgactc
 I  I  D  E  E  V  A  R  W  K  P  E  L  D  W  Y  K  E  R  L 
atgggcaagaaggtgtgcctatggcctggtggctccaaattgtggcattgggcgcacgtc
 M  G  K  K  V  C  L  W  P  G  G  S  K  L  W  H  W  A  H  V 
atcgaggaagaaatgggattgaaggtcgtgtccgtctacaccaagttcggacatcaaggc
 I  E  E  E  M  G  L  K  V  V  S  V  Y  T  K  F  G  H  Q  G 
gacatggagaagggaatagcccgatgtggcgaaggtactcttgcgatcgatgatccaaat
 D  M  E  K  G  I  A  R  C  G  E  G  T  L  A  I  D  D  P  N 
gaactcgaaggtttagaagctcttgagatgcttaaaccagatataattttgacgggaaag
 E  L  E  G  L  E  A  L  E  M  L  K  P  D  I  I  L  T  G  K 
cgtccaggtgaggtggcaaaaaaagtccgtgttccttacctcaatgcccacgcttaccac
 R  P  G  E  V  A  K  K  V  R  V  P  Y  L  N  A  H  A  Y  H 
aacgggccttacaaaggttttgaaggttgggtgcgttttgctagggatatttataatgct
 N  G  P  Y  K  G  F  E  G  W  V  R  F  A  R  D  I  Y  N  A 
atctacagtccaatccatcagctgtccggaatcgatataactaaggataatgcccctgaa
 I  Y  S  P  I  H  Q  L  S  G  I  D  I  T  K  D  N  A  P  E 
tgggggaatggctttcgtacgcgacagatgctcagtgacgggaatttgagcgacgctgtc
 W  G  N  G  F  R  T  R  Q  M  L  S  D  G  N  L  S  D  A  V 
aggaattcagaaacactgcgtcagtacacaggcggttatgactctgtatcaaaactgcga
 R  N  S  E  T  L  R  Q  Y  T  G  G  Y  D  S  V  S  K  L  R 
gaaagagagtatcccgcgtttgaacgtaaggttggtggtggctacccttacgacgttcct
 E  R  E  Y  P  A  F  E  R  K  V  G  G  G  **Y  P  Y  D  V  P** 
gattacgct
 **D  Y  A**

**SN371, CoxIV-Twin Strep-AnfG:**

atgctttcacttagacaatctattagatttttcaagccagctacaagaactttgtgttct
 M  L  S  L  R  Q  S  I  R  F  F  K  P  A  T  R  T  L  C  S 
tctagatatcttcttcagcaaaaaccttcagcatggagtcatcctcagtttgagaaaggt
 S  R  Y  L  L  Q  Q  K  P  **S  A  W  S  H  P  Q  F  E  K  G** 
ggaggttcaggtggtggaagcggtggatctgcttggtcacatccacaatttgaaaaagga
 **G  G  S  G  G  G  S  G  G  S  A  W  S  H  P  Q  F  E  K**  G 
ggtatgtccacggcctccgccgctgccgtagtcaaacagaaagtagaggcgcccgtacac
 G  M  S  T  A  S  A  A  A  V  V  K  Q  K  V  E  A  P  V  H 
ccgatggatgcgagaatcgacgaacttacagattacataatgaagaactgcctctggcag
 P  M  D  A  R  I  D  E  L  T  D  Y  I  M  K  N  C  L  W  Q 
ttccacagcaggagttgggacagggagaggcagaatgccgaaattctcaagaaaacgaaa
 F  H  S  R  S  W  D  R  E  R  Q  N  A  E  I  L  K  K  T  K 
gaattactttgtggcgagcctgtagatctatcaacatcacacgaccgatgctattgggtt
 E  L  L  C  G  E  P  V  D  L  S  T  S  H  D  R  C  Y  W  V 
gacgctgtctgcctagcggatgactaccgtgagcactacccgtggataaacagtatgtcc
 D  A  V  C  L  A  D  D  Y  R  E  H  Y  P  W  I  N  S  M  S 
aaggaggagataggaagtctaatgcaaggattaaaggaccgtatggactacttgaccata
 K  E  E  I  G  S  L  M  Q  G  L  K  D  R  M  D  Y  L  T  I 
acggggagcctaaatgaggagttatctgacaagcattac
 T  G  S  L  N  E  E  L  S  D  K  H  Y
